# Direct-to-consumer self-tests sold in the UK in 2023: cross sectional review of regulation and evidence of performance

**DOI:** 10.1136/bmj-2025-085547

**Published:** 2025-07-23

**Authors:** Bethany Hillier, Jonathan J Deeks, Joseph Alderman, Aditya U Kale, Trystan Macdonald, Simon W Baldwin, Katie Scandrett, Ridhi Agarwal, Alex Richter, Clare Davenport

**Affiliations:** 1 Department of Applied Health Sciences, School of Health Sciences, College of Medicine and Health, University of Birmingham, Birmingham, UK; 2 NIHR Birmingham Biomedical Research Centre, Birmingham, University Hospitals Birmingham NHS Foundation Trust and University of Birmingham, Birmingham, UK; 3 University Hospitals Birmingham NHS Foundation Trust, Birmingham, UK; 4 Department of Inflammation and Ageing, School of Infection, Inflammation and Immunology, College of Medicine and Health, University of Birmingham, Birmingham, UK; 5 Department of Clinical Immunology Services, School of Infection, Inflammation and Immunology, College of Medicine and Health, University of Birmingham, Birmingham, UK

## Abstract

**Objectives:**

To review the evidence base, clinical performance claims, and usability and safety of self-tests available for sale on the UK high street.

**Design:**

Cross sectional review of self-tests—regulation, evidence of performance, usability, and safety.

**Setting:**

Tests were identified from supermarkets, pharmacies, and health and wellbeing shops within a 10 mile radius of the University of Birmingham Edgbaston Campus in 2023.

**Main outcome measures:**

Accuracy claims of self-tests, samples used to derive accuracy measures, and regulatory requirements were summarised. Ergonomics, usability and safety concerns about the equipment and instructions, including interpretability and readability, were evaluated. Details of clinical and lay person study reports (population, sample size, reference or comparator tests, test process) were summarised, and methods were assessed using the Quality Assessment of Diagnostic Studies 2 (QUADAS-2) tool.

**Results:**

Thirty five self-tests were identified (30 obtained), which used seven different sample types and tested for 20 different biomarkers. Accuracy claims were made in instructions for use documents for 24/30 tests: accuracy for 19, sensitivity for 17, and specificity for 16. Performance claims of ≥98% were made on accuracy for 53% (10/19) of tests, 41% (7/17) on sensitivity, and 63% (10/16) on specificity. Where reference standards were reported in instructions for use documents, 29% (5/17) evaluated the accuracy of self-tests against similar rapid tests. For usability or safety, 18/30 self-tests had at least one high risk concern, 11 because of equipment, 10 because of the sampling process, and 15 owing to instructions or interpretation. Nine sets of clinical and lay person study reports were obtained (covering 12 tests). Across documents (nine clinical study reports and six lay person study reports) and QUADAS-2 domains, 73% were rated as having unclear risk of bias owing to poor reporting, and 58% were rated as having high applicability concerns because of inappropriate study designs. Participant descriptions were particularly inadequate in clinical study reports. Even within lay person study reports, few demographics (up to four) were presented. Some populations were unrepresentative of the intended user, inappropriate reference standards and thresholds were used, and mentions of blinding were scarce.

**Conclusions:**

This investigation highlights the need for improved regulatory oversight and clearer standards to ensure the safety and reliability of self-tests available on the UK market. Concerns about their ergonomics and usability might lead to test errors. Manufacturers' unwillingness to provide public access to study documents raises ethical concerns. Additionally, inadequate study design and reporting in available documentation hinders the ability to assess the evidence base supporting the use of self-tests. As the availability and use of self-tests continues to rise, improved regulatory oversight is urgently needed to protect the public from the effects of poor performing diagnostic self-tests.

## Introduction

Self-tests have more than a 50 year history of being used in the UK. The first self-test marketed for UK use was a pregnancy test in 1971,^1^ followed by the introduction of the first lateral flow test in 1985 (also a pregnancy test).^2^ Since then, self-tests for various conditions have become increasingly available.^3^ Their widespread use during the covid-19 pandemic in 2021 has increased public awareness with marketing in supermarkets and pharmacies in 2023,^4^ discussions on social media,^5^ as well as online and in the news.^6^

Concerns have been raised in medical settings about the impact of self-tests on patient decision making and demands on health services, particularly in primary care.^7^ Some self-tests are well established and have been endorsed by the NHS and the National Institute for Health and Care Excellence (NICE), such as pregnancy tests and blood glucose monitoring tests.^8^ ^9^ ^10^ ^11^ However, for most available self-tests, there is no current published guidance or endorsement on their use by NICE. Concerns exist about the accuracy claims of certain self-tests. For instance, a review of instructions for use (IFU) documents in 22 SARS-CoV-2 rapid tests compared with clinical studies included in a Cochrane review showed overestimation in sensitivity in IFUs by 12% (95% confidence interval 8% to 17%), with sensitivity overestimated by up to 45% in some tests.^12^

To be marketed in the EU and the UK, tests must obtain and then display CE marking with the identifying number of the EU notified body that reviewed it. The presence of CE marking might give consumers confidence that the test is accurate, safe, and reliable. EU national regulatory agencies devolve the responsibility to EU notified bodies to conduct regulatory assessments. A manufacturer chooses and pays the EU notified body to assess the technical documentation through a conformity assessment procedure,^13^ and to assess whether they meet the regulatory requirements set out by the In Vitro Diagnostic Medical Device Regulation (IVDR 2017/746).^14^ ^15^ Although the IVDR has superseded the In Vitro Diagnostic Medical Device Directive (IVDD 98/79/EC)^16^ ^17^ in the EU, its implementation has been delayed, and most tests have been sold under the IVDD regulations during this transition period. Across the UK, changes after Brexit have triggered a change to the medical device regulations for Great Britain,^18^ but Northern Ireland follows the same regulations as the EU.^19^ In time, tests in Great Britain will require UKCA (UK Conformity Assessment) marking rather than CE marking and will be assessed by UK approved bodies.^20^ ^21^ Great Britain currently accepts CE marking, which will continue until June 2030 while transitioning to the post-Brexit regulations.

The regulatory approach aims to ensure that the test is safe, and that its performance meets the manufacturer’s claims. All tests are expected to have a percentage of false negative (1−sensitivity) and false positive (1−specificity) results. It is important to use tests that are less likely to lead to these test errors, to have documentation that describes their performance limits, and to have mitigating strategies in place if considered necessary. The robustness of the claims of false negative and false positive fractions is driven by several factors, including the choice of clinical study design and its robust execution; the representativeness of the study group selected to use the test; the choice of an appropriate reference standard; the test being undertaken as it would in routine use; and the test’s ability to identify the relevant clinical condition. In developing the tests, laboratory analytical performance studies often overestimate the sensitivity and specificity of tests because technical experts run these tests on selected samples that are known to be positive or negative (based on other tests). Therefore, analytical performance studies are not sufficient to provide evidence for decision making by assessors and policy makers.^22^ The EU IVDR has drawn a clear distinction between analytical and clinical performance studies, and it is the responsibility of EU notified bodies to assess this.

Point-of-care tests used in medical practice are usually selected and interpreted by trained healthcare professionals. In contrast, the responsibility for choosing and interpreting the results of self-tests falls entirely on the user, who will often have no medical or laboratory expertise. Therefore, it is essential that the information, sampling, and test equipment are understandable, robust, and user friendly for the public; if not, risks of errors might affect their use, which could have a negative impact on health behaviours and might lead to harm. For example, false negatives could delay required treatment caused by false reassurance, and false positives could lead to anxiety, unnecessary follow-up tests and treatment, and could increase the burden on an already strained healthcare system.^23^ ^24^

We undertook a cross sectional review to assess whether self-test devices available for sale in UK high street shops are fit for their specified purposes, can benefit the public and reduce the health service demand, and are safe and reliable. Our paired paper^25^ identifies the claims of their intended use and the benefits and risks across the test-diagnosis-intervention pathway. Here, we report whether self-tests have performance that is known, adequate, and evidence based; provide evidence of unbiased studies to support their use; provide documents that are readable and understandable by members of the public; and whether the equipment, the sampling method, and the instructions are ergonomic, reliable, and free of error.

## Methods

### Sampling

Sampling aimed to give a comprehensive assessment of self-tests marketed in shops in the UK, while using a convenience sampling approach. Self-tests were identified that were on sale in the main UK supermarkets, community pharmacies, and health and wellbeing shops located within a 10 mile radius of the University of Birmingham Edgbaston Campus. This area encompasses largely urban areas of the metropolitan boroughs of Birmingham, Dudley, Sandwell, Solihull, and Walsall, with a combined population of approximately 2.6 million, representing 4.4% of the population of England and Wales.^26^ High street retailers selling self-tests were identified through an online business directory (yell.com). Only businesses with 10 or more UK outlets were selected as we considered this would likely provide a generalisable snapshot of nationwide availability. Self-tests were identified on the shelves of each store or marked as “available in-store” on the website; each outlet was visited and a single example of each available test kit was purchased. Sampling was conducted in April 2023.

Self-tests where the sample is intended to be taken, tested, and interpreted by the user were included. Other direct-to-consumer tests, such as self-sampling tests, where samples are taken by the user but sent to a laboratory for processing and interpretation, were excluded. Also excluded were pregnancy tests and ovulation tests because these are already established in community use; self-tests for detecting alcohol and drug misuse because they do not have the intended purpose of detecting or monitoring a disease or health condition; and test strips used as part of a test meter (as with a blood glucose monitor).

### Information sources and data extraction

For each test, we reviewed the equipment provided, the information on the packaging, the IFUs (sometimes labelled as patient information sheets or patient information leaflets), and any other documents included in the test box. We identified and tabulated the following data, which were extracted by one researcher and subsequently checked by a second researcher:

1. Characteristics of the biomarker, sample type, manufacturer, distributor (the company that buys tests from the manufacturer and sells them to pharmacies and stores), regulatory status, sellers, and costs.
2. Claims on the performance of the test detailed in the IFU or packaging.
3. Supporting evidence from reports obtained from the manufacturer’s registration file (also known as the technical file).
4. Aspects of the equipment, sampling, instructions and interpretation, and patient readability.

We requested reports from the distributor and manufacturer for each test from the manufacturer’s technical file (as used by the EU notified body to review the test before awarding CE marking), which included documents such as:

1. The IFU as agreed for the CE marking.
2. Clinical study reports (CSRs), which are full reports of clinical performance (accuracy) studies of the test.
3. Lay person study reports (LSRs), which are reports of studies that show the ability of these tests to be appropriately used and tested by lay people.

CSRs, which must be included in the technical file, refer to the reports that present performance evaluation data, with the self-testing device compared against a reference standard or comparator test. These data should originate from studies conducted in a clinical or other suitable environment. LSRs should present data on the handling, suitability, and usability of the device for its intended purpose of self-testing (ie, studies carried out with lay people), and the ease of understanding the labelling and instructions. CSRs and LSRs should also be provided for regulatory approval.^16^

Requests for reports and data were sent by personal email to all email and website links for manufacturers and distributors found in the packaging, the IFU, and websites for each test. The lead researcher (JJD) sent requests twice.

### Quality assessment of evidence documentation

CSRs and LSRs were assessed using the Quality Assessment of Diagnostic Studies 2 (QUADAS-2) tool.^27^ We applied the QUADAS-2 tool to all obtained CSRs and to any LSRs that reported accuracy metrics such as sensitivity and specificity. Quality assessment was independently conducted by two investigators (BH, JJD), with disagreements resolved through consensus.

### Evaluation of equipment, sampling process, and instructions

Coinvestigators (who included test experts, statisticians, clinicians, and a test manufacturer) met to review and evaluate the test, sampling equipment, and IFUs. Items that could cause test errors and practical challenges were identified and categorised according to the degree of risk or annoyance they might cause a user to experience when completing the test, receiving an incorrect result, or interpreting or acting upon it. Concerns were classified as high, moderate, or low risk.

Concerns were deemed to be high risk when the sampling process or its interpretation could potentially lead to erroneous results (eg, tests not being labelled as positive or negative, tests set at inappropriate thresholds, or colour charts containing errors). Concerns could be rated as high risk if there is a high probability of errors occurring, or when there is a probability of harm and that harm could be severe. Errors for tests assessing particularly consequential health risks such as cancer, blood glucose, HIV, and other infectious diseases were rated higher according to the IVDD regulations.^17^ Concerns were moderate risk when the test might fail with no result being possible (eg, insufficient amounts of equipment provided, uninterpretable, or potentially confusing instructions and figures).

Concerns were rated low risk when the equipment and information provided was inappropriate or inadequate (eg, mentioning that samples can be frozen, specifying the use of equipment not provided), but when a user could find a workaround (eg, the need to find a sterile pot for a urine sample), where the test or sampling was likely to cause frustration (eg, cassette windows being very small), or if the instructions did not make sense but could be implied (eg, no explanation on what a midstream urine sample was).

Assessments of concerns and errors were independently categorised by a subset of the team, consisting of clinicians and one test expert, followed by a consensus process to resolve disagreements. Descriptions of all concerns were documented and supplemented by photographs taken during the assessment process.

### Assessment of readability

Accessibility of the IFU documents was assessed by measuring readability using Readable software (https://app.readable.com), which calculates the number of letters, syllables, words, sentences, and paragraphs in each document. The Flesch reading ease scores (scored 1-100, higher scores indicate higher readability) and Flesch-Kincaid grades (scored 0-18)^28^ ^29^ were computed, together with counts of the number of words with three or more syllables. Flesch reading ease scores of 70-80 are expected to be analogous to US school grade 8 (school age 13-14, equivalent to UK school year 9).^30^ The control documents used to calculate readability measures were the UK Highway Code^31^ and books from the Harry Potter series^32^ ^33^ because most adults in the UK are expected to understand these books (around 85% of adults^34^). The first 1000-1500 words were selected from each control document. Type size was measured based on the first section of each IFU document.

### Patient and public involvement

Public engagement was conducted at a research showcase event at Queen Elizabeth Hospital, Birmingham, on 19 May 2023 where attendees were shown posters and packaging of self-tests we had purchased. Twenty members of the public and 30 healthcare professionals engaged and were asked about their awareness, experiences, trust in results, likelihood of using the self-tests, and the type of information they considered important to know about a test (supplementary table A1 lists questions asked). Insights from this exercise informed the data extraction framework.

## Results

We identified 35 different self-tests on sale in shops in the sample area in April 2023 (on the shelf or marked as “available in-store” on the website); 30 of these individual tests were obtained. The included tests have been labelled with identifiers, T1-T30; table 1 gives details of test information (supplementary table A2 provides further test characteristics). The 30 tests were from 14 different manufacturers, with up to nine tests per manufacturer. Three tests (T3, T26, T28) were close clones of other tests (T4, T25, T27), sold under a different name by a different distributor, but made by the same manufacturer with almost identical documentation.

**Table 1:** Test characteristics

Test ID	Test product name	Sample type	Manufacturer	UK distributor	Notified body
**T1**	Menopause Test	Urine	CARE diagnostica, Austria	SELFCheck	0483
**T2**	Flourish Menopause Test Kit	Urine	Veda-laboratory, France	Dendron brands	0483
**T3**	Menopause (FSH) Rapid Test	Urine	Hangzhou AllTest Biotech, China	Suresign	0123
**T4**	FSH Rapid Menopause Test Midstream	Urine	Hangzhou AllTest Biotech, China	Newfoundland	0123
**T5**	SP-10 Male Fertility Rapid Test	Semen	Hangzhou AllTest Biotech, China	Newfoundland	0123
**T6**	SpermCheck Fertility	Semen	PBM Princeton BioMeditech, USA	SpermCheck	1434
**T7**	SwimCount Sperm Quality Test	Semen	MotilityCount ApS, Denmark	SwimCount	2797
**T8**	SURE CHECK HIV Self-Test	Capillary blood	Chembio Diagnostic System, USA	Luas Diagnostics	0459
**T9**	Female Chlamydia STI Test Kit	Vaginal swab	CARE diagnostica, Austria	SELFCheck	0483
**T10**	Women's Intimate Self-test	Vaginal swab	Biosynex SA, France	Boots	2797
**T11**	Canestest Self-test for Vaginal Infections	Vaginal swab	Peptonic Medical Israel, Israel	Bayer	0483
**T12**	Urine Infection Test	Urine	CARE diagnostica, Austria	SELFCheck	0483
**T13**	Bowel Health Test	Faeces	CARE diagnostica, Austria	SELFCheck	0483
**T14**	FOB Rapid Test (Faeces)	Faeces	Hangzhou AllTest Biotech, China	Newfoundland	0123
**T15**	Prostate Health Test	Capillary blood	Veda-laboratory, France	SELFCheck	0483
**T16**	Stomach Ulcer Test	Capillary blood	Veda-laboratory, France	SELFCheck	0483
**T17**	Gluten Sensitivity Test	Capillary blood	Veda-laboratory, France	SELFCheck	0483
**T18**	One step Strep A Swab test	Throat swab (tonsils)	Guangzhou Wondfo Biotech, China	Not stated	0123
**T19**	Flowflex Influenza A/B Rapid Test (Self-Testing)	Nasal swab	ACON Biotech (Hangzhou), China	Newfoundland	0123
**T20**	Flowflex SARS-CoV-2 Antigen Rapid Test (Self-Testing)	Nasal swab	ACON Biotech (Hangzhou), China	Newfoundland	0123
**T21**	One step test for SARS-CoV-2 Antigen (Colloidal Gold)	Nasal swab	Getein Biotech, China	Every Genetic	1434
**T22**	STEPAHEAD COVID-19 Antigen Rapid Test Kit (Swab) For Self-Testing	Nasal swab	Safecare Biotech (Hangzhou), China	SHARE INFO	1434
**T23**	Microalbuminuria (MAU) Rapid Test Kit (Colloidal Gold)	Urine	Hangzhou Singclean, China	Newfoundland	0123
**T24**	TSH Rapid Test Cassette	Capillary blood	Hangzhou AllTest Biotech, China	Newfoundland	0123
**T25**	Ferritin Rapid Test Cassette	Capillary blood	Hangzhou AllTest Biotech, China	Newfoundland	0123
**T26**	Iron Deficiency	Capillary blood	Hangzhou AllTest Biotech, China	Suresign	0123
**T27**	Vitamin D Rapid Test Cassette	Capillary blood	Hangzhou AllTest Biotech, China	Newfoundland	0123
**T28**	Vitamin D Test	Capillary blood	Hangzhou AllTest Biotech, China	Suresign	0123
**T29**	Cholesterol Level Test	Capillary blood	CARE diagnostica, Austria	SELFCheck	0483
**T30**	Blood Glucose Test	Capillary blood	National Diagnostic Products, Australia	SELFCheck	0123

FOB=faecal occult blood; FSH=follicle-stimulating hormone; STI=sexually transmitted infection; TSH=thyroid stimulating hormone.

Half of the self-tests we obtained were from five manufacturers in China (15/30); the other tests were made by eight different manufacturers in Austria (5), France (5), the United States (2), Denmark (1), Australia (1), and Israel (1; table 1). Tests had CE marking by five different EU notified bodies, with most (24/30) regulated by two different EU notified bodies.

The 30 self-tests we obtained used seven different sample types and tested for 19 different conditions (or 20 biomarkers because two different biomarkers tested male fertility levels). Almost a quarter (23%, 7/30) of the self-tests could be deemed as moderate or high risk according to regulations^17^ or based on manufacturers’ claims. Under IVDD regulations,^17^ the HIV test (T8), the blood glucose test (T30), the chlamydia test (T9), and the prostate test (T15) would be classified as moderate or high risk. In addition, other tests described to help diagnose cancer might be classified as moderate risk by manufacturers (two bowel tests (T13, T14) and the stomach ulcer test (T16)).

### Instructions for use performance claims

Claims of test performance were stated in the IFU and on packaging (table 2). Of the 30 tests, 24 made claims of performance in the IFUs: 17 gave a sensitivity value, 16 gave a specificity value, and 19 included an “accuracy” or correlation value. No claims were made in IFUs for the remaining six tests: one reported reliability but not accuracy (T30); one reported the minimum quantity of the antigen the test is able to detect (ie, analytical sensitivity; not presented in table 2) but not clinical accuracy (T18); two stated accuracy on the box but not on the IFU (T5, T10); and two others made no statements (T26, T28). None mentioned predictive values, which would be a more useful measure for users, giving an indication of the post-test probability of them having the target condition. An IFU document for one test (T7) misinterpreted sensitivity and specificity as predictive values.

**Table 2:** Claims on performance and evidence from study instructions for use documents and packaging

Test ID	Test product name	No of samples	Group 1 (+)	Group 2 (−)	Accuracy, % (95% CI)	Sensitivity, % (95% CI)	Specificity, % (95% CI)	Claim on box	Comparator test (reference standard)	Participants or samples	Contact from manufacturer
T1	Menopause Test	65	—	—	—	97.14	93.33	Reliable	—	—	No response
T2	Flourish Menopause Test Kit	—	—	—	92.80	—	—	Reliable	—	—	Refused (M)
T3	Menopause (FSH) Rapid Test	250	85	165	100	100	100	Over 99% accurate	Another commercial test	Urine specimens	Provided (D)
T4	FSH Rapid Menopause Test Midstream	250	85	165	100	100	100	Over 99% accurate	Another commercial test	Urine specimens	Provided (DD)
T5	SP-10 Male Fertility Rapid Test	—	—	—	—	—	—	Over 98% accurate	—	—	Provided (D)
T6	SpermCheck Fertility	—	—	—	98	—	—	Proven accurate	Standard microscopic laboratory test	—	No response
T7	SwimCount Sperm Quality Test	—	—	—	95	96	91	—	Manual sperm count	—	Provided (M)
T8	SURE CHECK HIV Self-Test*	2554	503	2051	—	100 (99.1 to 100)	99.8 (99.5 to 100)	—	—	HIV-positive individuals; HIV-negative individuals; people from the US and the EU	No response
T9	Female Chlamydia STI Test Kit	596	—	—	—	85.70	98.30	Reliable	PCR method	—	No response
T10	Women's Intimate Self-test	—	—	—	—	—	—	Over 90% accurate	—	—	Refused (M)
T11	Canestest Self-test for Vaginal Infections	—	—	—	>90	91.8 (85.04 to 96.16)	92.9 (86.87 to 96.68)	More than 90% accurate	—	—	No response
T12	Urine Infection Test	—	—	—	—	Protein 91, nitrite 85, leucocytes 88	Protein 91, nitrite 99, leucocytes 95	Reliable	—	—	No response
T13	Bowel Health Test	138	—	—	—	91	—	Reliable	—	Participants scheduled for colonoscopy	No response
T14	FOB Rapid Test (Faeces)	464	64	400	99.10	—	—	Over 99% accurate	Another commercial rapid test	—	Provided (D)
T15	Prostate Health Test	—	—	—	Correlation 87% (81.82 to 92.33)	—	—	Reliable	Laboratory reference methods	Blood samples	Refused (M)
T16	Stomach Ulcer Test	—	—	—	≥90	—	—	Reliable	Reference laboratory methods	—	Refused (M)
T17	Gluten Sensitivity Test	—	—	—	97.00 (90.79 to 99.60)	—	—	Reliable	—	—	No response
T18	One step Strep A Swab test	—	—	—	—	—	—	—	—	—	No response
T19	Flowflex Influenza A/B Rapid Test	A 324, B 301	A 68, B 45	A 256, B 256	A 99.38 (97.63 to 99.98), B 99.67 (97.95 to 99.99)	A 100.00 (93.60 to 100.00), B 100.00 (90.62 to 100.00)	A 99.22 (97.01 to 99.97), B 99.61 (97.59 to 99.99)	—	SD Influenza A/B Rapid Test	Nasal swabs	Refused (M)
T20	Flowflex SARS-CoV-2 Antigen Rapid Test	605	170	435	98.8 (97.6 to 99.5)	97.1 (93.1 to 98.9)	99.5 (98.2 to 99.9)	—	RT-PCR	Nasal swabs from individuals suspected of COVID-19	Refused (M)
T21	One step test for SARS-CoV-2 Antigen	569	170	399	98.77 (97.48 to 99.40)	97.65 (94.11 to 99.08)	99.25 (97.81 to 99.74)	—	RT-PCR (Vitassay's qPCR kit)	569 individuals, 146 are asymptomatic	No response
T22	STEPAHEAD COVID-19 Antigen Rapid Test Kit	420	220	200	98.10	96.40	100	—	PCR	—	No response
T23	Microalbuminuria (MAU) Rapid Test Kit	210	125	85	98	98	97	Over 98% accurate	Quality control material	—	Provided (D)
T24	TSH Rapid Test Cassette	220	54	166	98.2 (95.4 to 99.5)	98.2 (90.1 to 99.9)	98.2 (94.8 to 99.6)	Over 98% accurate	ELISA	Whole blood samples	Provided (D)
T25	Ferritin Rapid Test Cassette	102	23	79	95.10	91.30	96.20	Over 95% accurate	A leading commercial CLIA test	Whole blood specimens	Provided (D)
T26	Iron Deficiency	—	—	—	—	—	—	—	—	—	Provided (DD)
T27	Vitamin D Rapid Test Cassette	90	4 and 56†	30	94.40	>99.9 and 94.6	93.3	Clinically tested accuracy	Predicate device (vitamin D rapid test)	Blood specimens	Provided (D)
T28	Vitamin D Test	—	—	—	—	—	—	Accurate	—	—	Provided (DD)
T29	Cholesterol Level Test	145	—	—	94	—	—	Reliable	Clinical laboratory Reflotron system	Blood samples	No response
T30	Blood Glucose Test	—	—	—	—	—	—	Reliable	—	—	Provided (M)

CI=confidence interval; CLIA=chemiluminescent immunoassays; D=from distributor; DD=from different distributor; ELISA=enzyme linked immunosorbent assay; FOB=faecal occult blood; FSH=follicle stimulating hormone; group 1=number of participants with positive or abnormal test result (determined by reference standard); group 2=number of participants with negative or normal test result (determined by reference standard); M=from manufacturer; PCR=polymerase chain reaction; RT-PCR=reverse transcription PCR; STI=sexually transmitted infection; TSH=thyroid stimulating hormone; qPCR=quantitative PCR.

* A second study was detailed in the instructions for use leaflet stating to evaluate the reliability of the HIV test (T8). This study involved 1885 participants altogether (395 positive and 1490 negative), all unaware of their HIV status, and participants performed the test on themselves. Sensitivity and specificity were not explicitly reported for this study, but can be deduced as 98.9% (from the true positive percentage) and 100% (from the true negative percentage), respectively. Self-test results were compared with results from “laboratory/reference methods.”

† Number of participants with deficient and insufficient vitamin D levels (determined by reference standard), respectively.

The statistical precision of estimates of sensitivity and specificity could be deduced in 12 of 30 tests based on statements of sample size or confidence intervals. Sample sizes alongside sensitivity statements were reported for 11/17 tests and confidence intervals for 6/17 tests. Sample sizes for positive or abnormal test results ranged from four (with a sample size of four deficient and 36 insufficient for the vitamin D test, T27) to 503 (the HIV test, T8). Sample sizes alongside specificity statements were reported for 11/16 tests, confidence intervals reported for 6/16 tests, and sample sizes for negative or normal test results varied between 30 (T27) and 2051 (T8). We were unable to reconcile the data in the table in the IFU for the microalbuminuria test (T23). Of the 19 IFU statements of accuracy, 12/19 stated total sample size and 6/19 included confidence intervals, while 5/19 made claims on accuracy without giving any information about total sample size or uncertainty. Total sample size ranged between 65 (T1) and 2554 (T8). Where claims were made, 98% accuracy or higher was claimed for more than half of tests (10/19), 98% sensitivity or higher was claimed for 41% (7/17), and 98% specificity or higher was claimed for 63% (10/16; table 2).

The reference standard or comparator test was stated in most (71%, 17/24) tests where claims of performance were made. None involved a clinical reference standard; four of 17 (24%) stated they used polymerase chain reaction tests (T9, T20-T22); seven (41%) stated a clinical laboratory method with no further detail; and five (29%) mentioned using a similar rapid test as a comparator test, with only one of these five (T19) naming the comparator. One (5.9%) stated that a quality control sample was used (T23).

The nature of the samples or participants evaluated was stated in 12 (50%, 12/24) tests where an accuracy claim was made. However, eight (67%, 8/12) described use of samples or specimens without any further detail of the source of samples or characteristics of participants. Four (33%) mentioned participants: one covid-19 test (T20) stated that they tested participants suspected to have covid-19; one covid-19 test (T21) was evaluated in mostly people with symptoms, stating 146/569 (26%) participants did not have symptoms; the HIV test (T8) indicated that the sample consisted of people who were HIV positive and HIV negative, from the US and the EU; and one bowel cancer test (T13) stated that the participants had been scheduled for colonoscopy.

A second study was detailed in the IFU for the HIV test (T8), stating it evaluated its “reliability.” This study involved participants unaware of their HIV status, the participants performed the test on themselves, and the self-test was compared against “laboratory/reference methods.” However, the sensitivity and specificity metrics presented in the IFU are not obtained from this study, but from the study described in table 2, which provided very little detail and potentially incorporated a two gate study design.

### Ability to access regulatory documents

We asked manufacturers and distributors to share CSRs and LSRs used by EU notified bodies to validate the performance claims of the self-tests. We obtained nine sets of reports (for T4, T5, T7, T14, T23-T25, T27, T30), which cover 12 tests in total (as T3, T26, T28 are clones of T4, T25, T27; supplementary table A3). Seven document sets were provided by one distributor, which sold tests from two manufacturers, all regulated by the same notified body. Documents for tests T7 and T30 were provided by manufacturers (T7 provided an FDA (US Food and Drug Administration) regulatory document).

Of the 10 other manufacturers, three stated that their reports were commercially confidential and refused to provide the performance documents, one of which (based on a telephone conversation with the lead investigator (JJD)) was concerned about how we might use the information publicly. These three companies manufacture seven of the 30 tests. No communication was received from the other seven manufacturers, covering 11 tests.

### Clinical and lay person study reports

**Fig 1:**
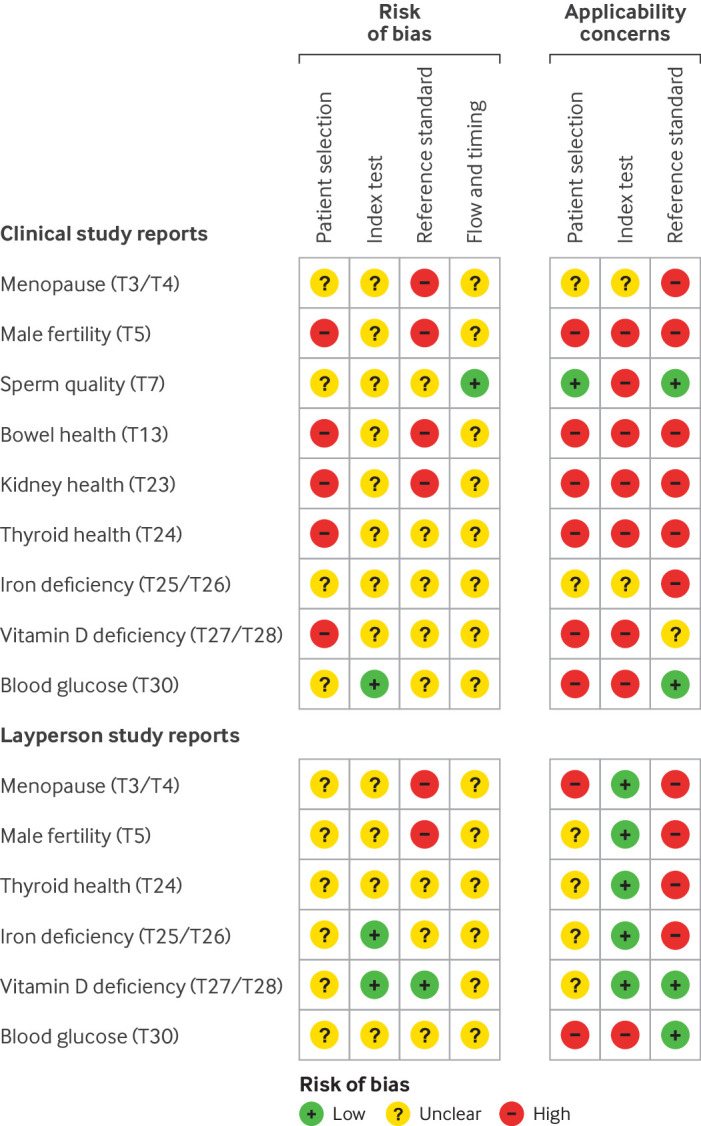
QUADAS-2 (Quality Assessment of Diagnostic Studies 2) summary figure of risk of bias and applicability concerns

Out of the 18 documents accessed (from 12/30 tests), the methodological quality assessment focused on 15 documents (nine CSRs and six LSRs) that included test accuracy claims. The reports were critically reviewed using the QUADAS-2 tool,^27^ which groups the risk of bias and applicability concerns under four domains: patient selection; index test; reference standard; flow and timing (fig 1).

Of the 15 documents, 15 (100%), 12 (80%), 14 (93%), and 14 (93%) were rated as having unclear or high risk of bias in each of the four domains, respectively; 14 (93%), 10 (67%), and 11 (73%) were rated as having unclear or high concerns about applicability in the first three respective domains. Across the documents and domains, the risk of bias was rated as unclear for 73% of judgments owing to the lack of reporting information of the study methods. Applicability concerns were rated as high for 58% of judgments because it was clear that many aspects of the studies did not match the participants, the use of the index test, and the appropriate choice of reference standard. Below, we highlight examples of high and low risk of bias shown across the CSRs and LSRs.

#### Patient selection

The origin (ie, setting) and country were described for around half (56%, 5/9) of the CSRs, and only two (22%) described any participant characteristics. Semen samples were collected from a hospital for one male fertility test (T5) and from fertility clinics or a sperm donor bank for another (T7); however, age categories were the only personal details given for test T7 and no demographics were given for test T5. Urine samples were collected from a hospital for the microalbuminuria test (T23), capillary blood samples were collected from patients attending a diabetes clinic for the blood glucose test (T30), and urine samples were collected from “patients who are under outpatient service, emergency treatment, and [inpatients]” for the menopause test (T3/T4), but the CSRs provided no further details on the sample population. Other CSRs provided no details on the origins, demographics, or clinical status of samples, or whether the samples were from unique patients or included several samples for each patient.

Most LSRs (78%, 7/9) provided few participant characteristics, with 22% (2/9) not describing participants at all. However, the population for one menopause test (T3/T4), for which the most demographic characteristics were reported, was not representative of the intended user of the self-test, raising high applicability concerns. Details were provided on four demographic variables: age group, time since last menstrual period, education level, and sex (all female). The included women (n=105) were unrepresentative of those likely to purchase a menopause test, with 59% (62/105) aged 20-40 years (supplementary figure A1(a)), and with 25% (24/95) and 62% (59/95) stated to have had their last menstrual period in the past one or two months, respectively (supplementary figure A1(b)). Menopause usually affects women aged between 45 and 55 years, with the average age for menopause in the UK being 51 years.^35^

Additionally, from the data provided within CSRs on the reference or comparator tests, the distribution of concentrations measured by the ferritin laboratory CLIA (chemiluminescent immunoassay) test (T25/T26), the thyroid stimulating hormone laboratory ELISA (enzyme linked immunosorbent assay) test (T24) and the quantitative vitamin D rapid test (T27/T28) had reduced frequencies close to the cutoff values (supplementary figure A1(c-e)). This pattern in the distributions could indicate that selective patient inclusion was implemented, which might have led to an overestimation of the accuracy of the self-tests in question^36^ ^37^ ^38^; however, this cannot be confirmed given the lack of information provided in the CSRs. Biomarker values that lie close to disease thresholds are more likely to be misclassified as false positives and false negatives compared with those that have more extreme values.

#### Index test

No CSRs mentioned using blinding to conceal the results of the multiple tests that are compared. For some tests, samples were tested across several formats (eg, dipstick, cassette, midstream) and test batches, and test results were judged visually, which precludes result concealment. The LSR for the vitamin D test (T27/T28), conducted by an independent unit, detailed sampling and ensured blinding, with comparisons to venous samples. Other LSRs lacked comparable rigour, providing no details on sampling methods, test locations, or blinding. However, most LSRs (83%, 5/6) were rated as having low applicability concerns for the index test domain because the test appears to have been conducted as intended by lay users. The blood glucose test LSR (T30) was rated as having high applicability concerns because the users received training in the use of the test strips beforehand.

For most CSRs (78%, 7/9), testing of the index test appears to have been undertaken by laboratory technicians, not lay users. Additionally, despite the blood glucose self-test (T30) being sold as test strips with results visually inspected against a colour card, the CSR evaluated the test by using digital Roche meters. Because of poor reporting in the other two CSRs (T3/T4 and T25/T26), it is unclear who undertook the testing. From the menopause test CSR (T3/T4), it seems that seven separate tests were conducted on the same single urine sample, which seems unlikely to have been undertaken by a lay user.

#### Reference standard

Almost half of CSRs (44%, 4/9) stated that a similar rapid test was used as the reference standard, one reported the use of a quantitative rapid test (T27/T28), and none were compared against a clinical reference standard. Two CSRs (22%; T24, T25/T26) evaluated self-tests against laboratory assays (ELISA or CLIA), but did not clarify whether they used capillary samples (as in the self-test) or venous samples, which require venipuncture by a professional. In one CSR, the blood glucose test (T30) was evaluated against capillary samples analysed with a laboratory based technique, YSI 2300 (the YSI 2300 STAT PLUS Glucose Lactate Analyzer is an FDA cleared method that was the most widely used reference standard or comparator test for determining the accuracy of blood glucose measurement products during its production). In another CSR, a male fertility test (T7) used microscopy based manual sperm counts. Blinding against index test results was not mentioned in any CSRs. Applicability concerns were high for most CSRs (67%, 6/9) in the reference standard domain owing to similar rapid tests being used for four and (despite using laboratory based reference standards) a single threshold was used regardless of patient characteristics for the thyroid health test (T24) and the iron deficiency test (T25/T26).

Blinding against index test results was not mentioned in LSRs, except one (5/6); the vitamin D test LSR (T27/T28) clearly stated that the sample was tested by a point-of-care operator who had not observed the self-test result. Similar rapid tests were used as the reference standard for two LSRs (T3/T4, T5), raising high applicability concerns. The remaining LSRs (T24, T25/T26, T27/T28, T30) used laboratory based reference or comparator tests, however applicability concerns remained high for another two tests (T24, T25/T26) owing to the use of a single threshold for all participants, irrespective of their characteristics. Further details about mismatches between the claimed clinical conditions and the biomarker being measured are covered in our paired paper.^25^

#### Flow and timing

Flow and timing risk of bias was deemed to be unclear for most CSRs and LSRs (93%, 14/15). This judgment was made because of poor reporting of the time interval between tests and unclear descriptions of the analysis process, particularly whether any samples were excluded or if results were presented for each participant (ie, whether there were several samples for each participant).

#### Usability assessments

All nine LSRs recruited volunteers or lay users. In seven LSRs, ease-of-use assessments were conducted that involved subjective ratings, not clearly described: all assessments were rated between 97% and 100% (median 100%) positive for manufacturer studies, and rated between 82% and 100% (median 94%) in the one independent study for the vitamin D test (T27/T28). Subjective assessments included how easy it was to understand the IFU and the ease of conducting the test processes, such as sampling and reading results.

The LSR for the microalbuminuria test (T23) reported assessments from 100 volunteers, but findings were from 25 volunteers testing personal samples and 75 testing “quality control materials” (samples spiked with the biomarker). The LSR for the glucose test (T30) trained users to visually interpret results, which were compared with technician performed YSI 2300 analyses, with no usability assessments documented. The LSR for the sperm quality test (T7) evaluated usability at home, with photo based test interpretations cross checked by professionals. Participants rated the IFUs, sample handling, and ease of interpretation, but blinding and independent oversight were not included.

### Issues, errors, and concerns about usability

**Fig 2:**
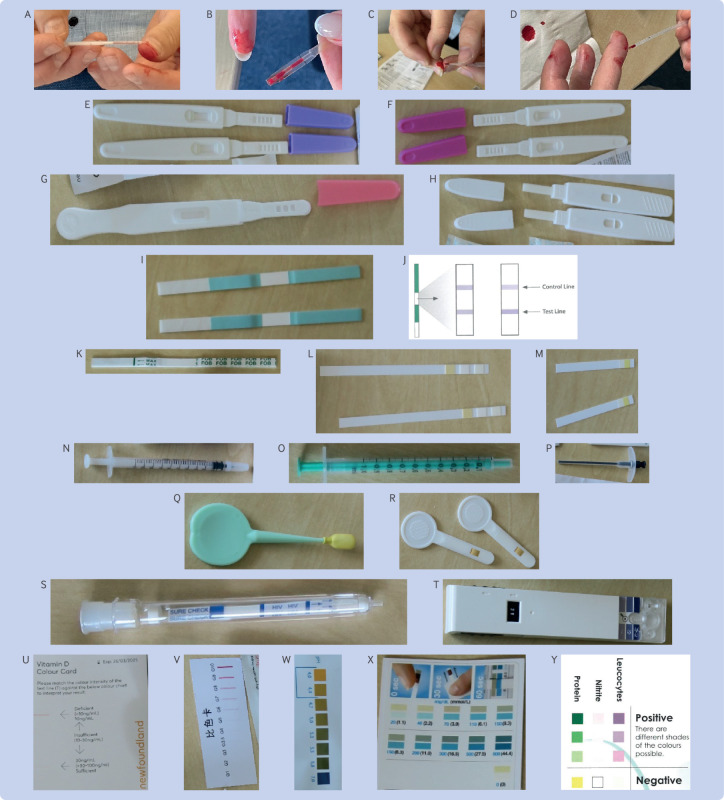
Examples of device and sampling methods. (A-D) Pipette examples from T15-T17, T24-T28. (E-H) Cassettes from menopause tests (T3-T4), microalbuminuria test (T23) and another menopause test (T2), respectively. (I) Dipstick from menopause test (T1). (J) Diagram of dipstick and colour chart from menopause test (T1). (K-M) Dipsticks from faecal occult blood test (T14), urine infection test (T12), and blood glucose test (T30), respectively. (N-P) Syringes from Newfoundland male fertility test (T5), SwimCount sperm quality test (T7), and SpermCheck male fertility test (T6), respectively. (Q, R) Vaginal swabs from Canestest self-test for vaginal infections (T11) and Boots women’s intimate test (T10), respectively. (S, T) Test devices from HIV self-test (T8) and SwimCount sperm quality test (T7), respectively. (U-Y) Colour charts from Newfoundland vitamin D test (T27), microalbuminuria test (T23), Boots women’s intimate test (T10), blood glucose test (T30), and urine infection test (T12), respectively

**Fig 3:**
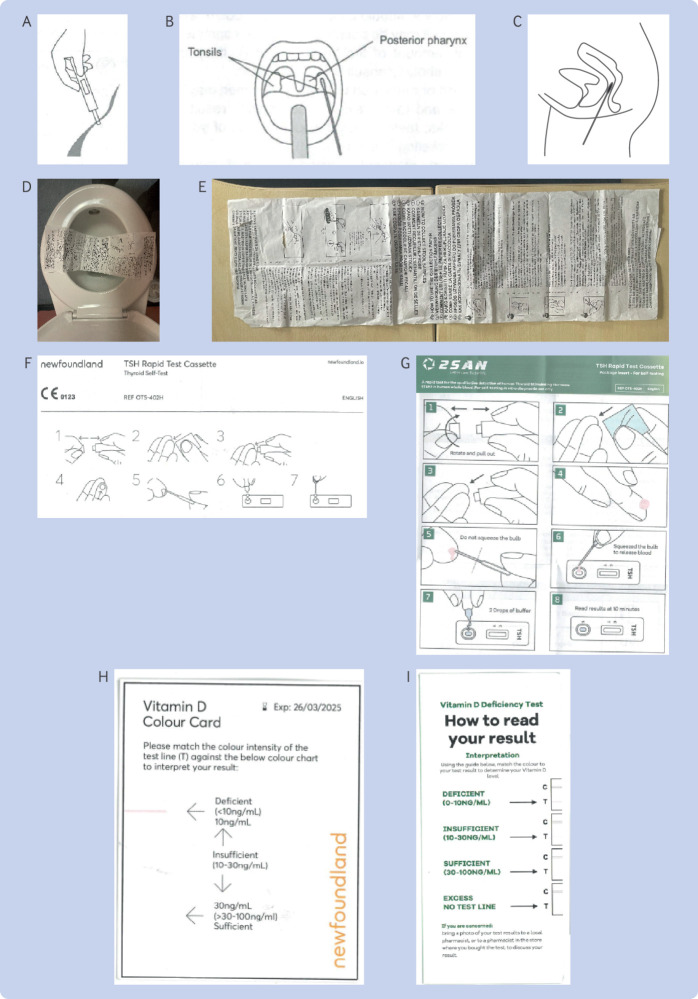
Examples of instructions and figures from instructions for use (IFU) documents. (A) Diagram of taking a midstream urine sample in IFU for menopause tests (T3, T4), and microalbuminuria test (T23). (B) Diagram of taking throat sample (tonsils) in IFU for Strep A test (T18). (C) Diagram of taking vaginal swab in IFU for female chlamydia test (T9). (D) Instructions document for faecal occult blood test (T14), placed on a toilet seat as instructed for collecting faecal sample. (E) Instructions document for faecal occult blood test (T14). (F, G) Pictorial instructions of how to take capillary blood sample in IFU for thyroid test from different distributors: Newfoundland (T24) and clone test from 2San (not included in set), respectively. (H, I) Colour charts for reading results in IFUs for vitamin D test from same manufacturer but different distributors: Newfoundland (T27) and clone test from 2San (not included in set), respectively

Our assessment of the usability and correctness of the equipment, sampling, and instructions raised concerns for 26 of the 30 tests, of which 18 were rated as having high risk because the probability of an error was high or the potential harm was severe. The three covid tests (T20-T22) and the influenza test (T19) raised no concerns. The sperm quality (T5-T7), vaginal infection (T10-T11), cholesterol (T29), and blood glucose (T30) tests only had moderate or low risk concerns (see supplementary tables A4 and A5, with illustrations in fig 2 and fig 3). High risk concerns were noted about the equipment (11 tests, 13 concerns), sampling (10 tests, 10 concerns), and instructions and interpretation (15 tests, 34 concerns). Eleven tests had three or more high risk concerns (T1, T12, T14, T15, T17, T23-T28).

The microalbuminuria test (T23) had the highest number of total concerns (13). The menopause tests (T1-T4) had seven, four, eight, and ten concerns, respectively; the faecal occult blood test (T14) had seven; the thyroid test (T24) had six; the two vitamin D tests (T27, T28) had six; the iron deficiency tests (T25, T26) had four and five, respectively; the prostate cancer (T15), bowel cancer (T13), chlamydia (T9), and gluten tests (T17) all had four concerns; and the HIV test (T8) had two. We categorised the type of issues observed into 10 groups (A-J), with a miscellaneous (K) set. Groups A-E relate to equipment, groups F-H relate to sampling, and groups I-K relate to interpretation or instructions.

High risk concerns in equipment include (A) dipsticks having no orientation mark (potentially resulting in people using the wrong end of the dipstick (T1); fig 2I); (B) no labelling of T (test) and C (control) marks on the dipstick or the instructions, and so the results could be read wrong (T1, T8, T9, T14; fig 2I, K, S); (C) no sterile pot provided in an infection based test (T12); (D) marks on lateral flow test cassette not being explained (T15-T17); (E) errors in the labelling of the thyroid pipette within the IFU (T24), which meant it was not clear how much blood should be collected (fig 3F, G).

The sampling process (F) for using plastic pipettes to collect capillary finger prick samples was regarded as a high risk concern in eight tests because it did not guarantee adequate blood samples (T15-T17, T24-T28). Five study team members tried to follow the instructions to collect these samples, but only one was successful, with inadequate blood volumes, samples quickly congealing, and bubbles in the pipette (fig 2A-D). The sampling instructions (G) were inadequate or challenging: the direction for the chlamydia test (T9), to “swab through the vagina as far as the neck of the womb” (fig 3C), could risk false negatives if self-sampling was inadequate; visual directions on how to collect a midstream urine sample (H) required for a bacterial test (T1, T3/T4, T23) were inadequate.

Interpreting the results for 10 (T13-T15, T17, T23-T28) of the 30 tests caused issues in (I) inappropriate choice, description, or poor use of thresholds because the test must be interpreted in accordance with a reference range (typically based on age, condition (eg, pregnancy), sex) or the stated threshold does not match the threshold used in routine clinical practice or guidelines. An example is the prostate test (T15) where a prostate specific antigen value >2.5ng/mL is abnormal in people aged 40-49 years, but <6.5 ng/mL is normal in those aged 70-79 years.^39^ The use of a fixed lateral flow test in these situations can lead to wrongly misclassifying results as positive or negative. Visual challenges (J) were observed in instructions with unclear labels in colour charts, particularly for semiquantitative tests (especially for people with colour blindness (fig 2U-Y); T1, T2, T7, T10, T12, T23, T27-T30); and errors in colour charts (fig 3H, I; T27, T28).

Miscellaneous concerns or errors (K) in interpretation include confusion when T lines on lateral flow tests indicate normality, reversing the logic of what is meant to be abnormal (T6, T23); failure to explain the meaning of a negative result (T3, T4); both coloured or clear T lines indicating a negative test (T1-T4); errors in the instructions where the figures do not match (T24); text wrongly stating only two blood drops should be used when a full pipette is required (T24); instructions mixing the order of buffer or blood sampled to be added to the lateral flow test (T24; eg, fig 3F, G show two different versions of the IFU of the same test from two different distributors that show contradictory instructions). One faecal test provided instructions that were printed on the sling used to collect the faecal sample—these therefore become unreadable during the sampling process because the sling must be attached to the toilet seat (fig 3D; T14). Several urine tests gave instructions for freezing samples (down to −20°C; T4, T23), to use a centrifuge if samples are cloudy (T3, T4, T23), or because specimens might be infectious and be a potential biological hazard, to wear disposable gloves and masks to prevent contact (T23)—all equipment that is expected in a laboratory test, and not usually available in a home setting.

### Readability

IFUs of 10 of the 30 tests had Flesch-Kincaid reading grades of 8 or lower, indicating they were appropriate for people with reading levels of typical 13-14 year olds or younger (supplementary table A6). The IFUs for the other 20/30 tests had Flesch-Kincaid reading grades of 9-10, with 17-20% of words having three or more syllables, including several medical and technical terms. Assessment of the control documents showed Flesch-Kincaid grade 6-7, with the proportion of words with three or more syllables between 4% and 10%. All self-test IFU documents were found to be harder to read than the control documents.

EU guidelines for labelling and packaging leaflets of medicinal products state a type size of nine points as a minimum.^40^ Only three of the 30 tests met this requirement; half (15/30) were printed in type sizes less than seven points (supplementary table A6).

## Discussion

### Statement of principal findings

The evidence base for the accuracy claims of self-tests available on the UK market is largely not publicly available. Three manufacturers refused to provide us with the requested documents. The reasons for rejection were that the information is commercially confidential or sensitive, and they were concerned that we would share the information publicly. Only one distributor and two manufacturers provided us with CSRs and LSRs, and so we had documentation for less than half (12/30) of the self-tests obtained. After the project, the head of journalism at *The BMJ* sent the same requests to the non-responders with the same result. Although it is not required by law for the companies to provide us with these documents, manufacturers should be encouraged to be transparent with their findings and regulations should be amended to ensure all research is made publicly accessible.

Even when we were able to access the study reports behind these accuracy claims, the studies were of low methodological quality. In comparison to established STARD (Standards for Reporting of Diagnostic Accuracy Studies) reporting guidelines for test evaluation research,^41^ ^42^ there was insufficient information on various aspects of the studies, such as recruitment methods, processes of the self-test and reference standard or comparator tests, and the populations for which the self-tests were evaluated, potentially introducing selective publication bias.^43^

Participants were described poorly in CSRs, often merely referred to as “samples” or “specimens,” with no reference to people, which makes it difficult to identify whether there were several samples from the same people. The studies described in the CSRs were conducted in laboratory settings (by technicians and not the intended users), and two gate studies were implemented for some. CSRs are labelled as “clinical” studies, but most are analytical performance studies, as shown by the methods reported. When populations were described, such as in the LSRs, descriptions were limited and, in some cases, unrepresentative of the intended user. The distribution of biomarker concentration levels indicated that selective patient inclusion (ie, excluding values around the test threshold) might have occurred, which can lead to an overestimation of accuracy.^36^ ^37^ ^38^

Overestimation of accuracy can occur when using a comparator or reference standard test that is very similar in its mechanism to the self-test being evaluated (and has positively correlated errors), rather than against a clinical reference standard.^36^ ^37^ ^44^ Comparing one imperfect test with another will only assess the agreement between these tests and will not assess the true accuracy of the index test. A similar rapid test was used as a comparator for four CSRs (44%, 4/9). Some CSRs (44%, 4/9) reported the use of laboratory based reference standards, but there were concerns about inappropriate thresholds for half of these. There was also no mention of blinding of the index test result for any CSRs.

Some IFU documentation and test packaging (for 11/30 tests) only reported or emphasised being “accurate” or reliable, without distinguishing between sensitivity and specificity, leaving the reader unable to consider the separate probabilities of false positives and false negatives. These errors can have very different harms and consequences, so it is important to be able to differentiate between them.

For a large number (18/30) of the self-tests, we observed high risk concerns about at least one of the following: equipment, sampling, instructions, or interpretation. These were rated as high risk because the probability of the error could be high, or the impact of the error could be severe. From the readability assessment, there were concerns about the use of small type sizes and the length of words and sentences. Further work into the content of the IFUs is discussed in our paired paper^25^ about whether the healthcare advice is reliable and aligns with national guidelines.

We observed higher quality reports for some tests, such as the vitamin D test LSR (T27/T28). It is possible that some of the tests, for which we could not obtain CSRs and LSRs, might have strong evidence supporting their use, but without any documentary evidence available, we cannot draw any conclusions about their test performance.

### Strengths and weaknesses of the study

We have undertaken a robust, reliable evaluation of a sample of self-tests available to the UK public. We have engaged with experts to assess the multidisciplinary aspects of self-tests, including regulators and regulatory experts, clinical and public health experts, immunologists, clinical epidemiologists, statisticians, and a test manufacturer. Despite achieving consensus, there remains some subjectivity to elements of our assessment, particularly of concerns or errors, and while others might reach different conclusions on some aspects, we have provided full documentation (in supplementary appendix tables) so that the source data are accessible to check our decision making.

The study sample was restricted to the Birmingham area partially for reasons of convenience, but also because the area includes stores for most of the UK supermarkets, corporate pharmacies, and health and wellbeing shops, and so are probably indicative of the full range of self-tests available across the UK high streets. Our sample did not include self-tests sold only in independent pharmacies (which have 25% of the market share of community pharmacies), but it is unlikely that the self-tests they sell are different from those sold in corporate pharmacies.

Our assessments have been restricted by the lack of access and poor reporting of documentation provided by manufacturers, which we report as a critical finding. We have only been able to comment on documents we were able to access, and while studies with better study design and reporting might exist, these are also not accessible to the public.

We did not use the tests we purchased to assess their accuracy directly because this was not intended as part of our project and would have required undertaking new primary studies for each of the tests. Additionally, we have not undertaken lay person usability studies, including readability assessments. Our approach to assessing readability is a numerical based analysis of the text, which provides a proxy assessment for readability. Our text and appendix tables provide direct quotes from IFU documents to provide a flavour of their content, but future work involving the public in designing and testing self-test IFUs is planned.

Our initial sample of tests was obtained two years ago. We have been monitoring the market and have noticed that most of the tests in the current cohort are still available, but with many others added. In a search undertaken in December 2024, we identified 63 self-tests—approximately double the number of self-tests available compared with our original search. Many of the new tests are clones of tests already in our cohort (but sold under different names and by different distributors). Only two of the new tests claimed to test for a different condition than the original set: an allergy test and an ovarian reserve test (but using the same biomarker as the menopause tests).

### Explanations and implications for the public, clinicians, and policy makers

Our observation that a reliable evidence base is lacking for most self-tests in our sample raises concerns about the attention manufacturers and regulators give to the importance of scientific integrity, transparency, reliability, and ethical principles. Inaccurate tests, or tests used incorrectly, could cause harm, misleading people as to whether or not they have a condition. The harm is most likely to be indirect and not obvious or immediate, but it could lead to wrong decisions about treatments and disease or condition management, and psychological harm owing to misinformation. People might receive further tests unnecessarily, which could be invasive, expensive, or involve exposure to radiation; or people might be falsely reassured that they do not have a condition, leading to delayed or missed diagnoses, which could affect long term prognosis and delay treatment.

As with drugs, it is essential to have full access to study reports to make informed decision making on the use of diagnostic tests. With no ability to check and understand the study designs and findings of the marketed self-tests, we are not able to recommend their use as part of health self-management by the public and clinicians. It is important that consumers can access the right information to make informed decisions around the purchasing of direct-to-consumer tests. Under the new EU IVDR regulations, it is required that a Summary of Safety and Performance (SSP) document is “made available to the public” through an online database (EUDAMED).^45^ ^46^ This documentation should summarise the evidence and studies behind the performance claims of medical devices, including self-tests. Forthcoming UK regulations have the opportunity to adopt a similar requirement.

We observed a systematic unwillingness among manufacturers to provide the evidence base for use by clinicians, patients, and the public. Under the previous IVDD regulations in the UK and EU, there was no legal requirement for manufacturers or notified bodies to make the results of test studies public when awarding CE marking. While the manufacturers have not acted unlawfully in refusing to provide their reports, this system is in contrast to the ethical principles embedded in the Declaration of Helsinki: “Researchers have a duty to make publicly available the results of their research on human participants and are accountable for the timeliness, completeness, and accuracy of their reports.”^47^ Although manufacturers and developers might claim that internal development work does not constitute conducting “research,” should companies undertaking such work not be held to same ethical standards? The new requirement for public SSP documentation represents a step in the right direction towards greater transparency in the research of medical devices.

The reporting of clinical studies could be improved by manufacturers following the STARD reporting guideline.^41^ ^42^ However, the reports that we did access show that many, but not all, self-tests have been regulated on the basis of analytical evidence from laboratory studies and not clinical evidence obtained from using the tests in the people, setting, and manner they were intended for. Although studies were labelled as being “clinical” study reports (or variations thereof) by manufacturers, this is largely a misnomer. Rather than estimating clinical accuracy, studies of this nature estimate analytical accuracy—the performance of a test under strongly controlled conditions, which is compared with results for a similar laboratory analyte rather than with a clinical diagnostic reference standard. The Royal Statistical Society working group on diagnostic tests stated that “Analytical performance provides necessary but insufficient evidence to implement in vitro diagnostics”^22^; they help to assess whether a test could measure the agreement with a matching reference analyte, but not whether its use leads to a clinical diagnosis in a relevant patient.

The very high accuracy claims in the self-tests are largely those of analytical accuracy in a laboratory setting compared with a laboratory comparator, not the experience of a lay person using the test for self-diagnosis of a clinical condition. The Royal Statistical Society working group stated that “Field or clinical evaluation studies are needed to evaluate the performance of an in vitro diagnostic for each intended use; where the intended use specifies (a) the people, place and purpose of testing; (b) the target condition that testing aims to detect; (c) the test’s specimen type and how the specimen is taken, stored and transported and by whom; and (d) details of the individuals, training and facilities where testing is done.”^22^ Assessments of the clinical studies need to follow these requirements to provide relevant and informative statements for these tests.

It is essential that self-tests are well designed and follow the principles of ergonomic design to make sure they perform properly through the stages of obtaining a sample, execution of the test and interpretation of the results, by members of the public and clinicians. We observed many issues with the test sampling (ie, specimen collection) and testing equipment that could have led to failures or errors. As the choice, sampling, execution, interpretation, and decision making in a self-test are entirely the responsibility of the user, clear instructions are critical and need to be thought through with the same concern as with an over-the-counter drug. There was little evidence of the content, wording, and presentation of IFUs being well designed with patient testing. Our team's observation (which included several clinicians) was that many of the IFUs were not fit for purpose, with misleading, inappropriate, and occasionally erroneous text.

The regulatory assessments conducted by EU notified bodies for our sample of devices has raised some concerns. The EU notified bodies act as checkers before the sale of self-tests, and are charged with assessing whether the tests meet required safety and performance standards. The current assessment process for self-tests is not easy to follow and open to subjective interpretation. It is unclear how EU notified bodies are monitored to show that their assessments are consistent and appropriate. This study has highlighted many issues that raise questions about whether the framework is appropriate.

We shared the findings of our report with the Medicines and Healthcare products Regulatory Agency (MHRA) at the earliest opportunity, who have taken note with concern and are taking action. Following Brexit, the MHRA has the opportunity to change the process from CE marking to UKCA marking, with UK conformity bodies (called UK approved bodies) being the assessors of self-tests. Regulators in other jurisdictions should also take note of these findings. Although there are many opportunities for self-tests to benefit the public, realising these benefits requires effective regulatory oversight and frameworks to ensure that the public are not placed at undue risk of harm.

### Conclusions

Unreliable, inaccurate, and unfit-for-purpose tests can lead to incorrect test results, and can cause various harms, such as patient anxiety, increased burden on healthcare systems and general practitioners (because of false positive results), or delayed treatment and false complacency or reassurance (because of false negative results). Concerns over the ergonomics and usability identified in this review could lead to test errors and therefore might indirectly cause harm to the public. The unwillingness of manufacturers to provide access to study documentation raises ethical concerns, and concerns over their claims. Furthermore, the insufficient attention to study design and reporting standards in the available documentation has hindered the ability to assess the evidence base supporting the use of self-tests. This review highlights the need for clearer standards to ensure the safety and reliability of self-tests on the UK market. As the availability and use of self-tests continues to rise, improved regulatory oversight is urgent to protect the public from the effects of unreliable tests. Although most self-tests meet the standards, the rise in poor performing self-tests must be recognised for consumers to be aware of the tests they are purchasing and the purpose they serve in self-diagnosis.

**What is already known on this topic**

The availability of self-tests on the UK market has increased since the covid-19 pandemic, with tests for many more conditions available, including high risk conditions such as HIV and cancer
Self-tests in the UK are assessed by designated regulatory bodies (EU notified bodies or UK approved bodies), but they have not yet been systematically reviewed by researchers or UK government bodies
Self-tests with CE marking must meet the new EU In Vitro Diagnostic Regulations (transitioning from In Vitro Diagnostic Directive regulations at time of publication); in Great Britain, self-test regulations are currently undergoing a post-Brexit update

**What this study adds**

The evidence base for the accuracy claims of self-tests available on the UK market is largely not publicly available, leading to uncertainty in their performance claims
Many self-tests in the UK have been approved by EU notified bodies based on analytical evidence from laboratory studies with poor descriptions of study populations, unrepresentative populations, unsuitable choices of reference or comparator tests, and lack of blinding
Issues with sampling and test equipment were observed that might lead to errors; the responsibility of all stages of using self-tests lies entirely with the user, therefore clear and well considered instructions are critical

## Data Availability

No additional data available.

## Web extra

Extra material supplied by authors

Web appendix: Appendix tables

## Notes

Contributors: The project was conceptualised by JJD, AR, and CD. JJD obtained funding, supervised and managed the project. BH, JJD, AR, and CD designed the study and its methodology. BH, JJD, SWB, KS, RA, AR, and CD curated the data. BH, JJD, JA, AUK, TM, SWB, KS, RA, AR, and CD took part in the formal analysis, investigation, and writing the original draft, and reviewing the final manuscript. BH and JJD are joint first authors. AR and CD are joint last authors. JJD acts as guarantor. The corresponding author attests that all listed authors meet authorship criteria and that no others meeting the criteria have been omitted.

Transparency: The lead author affirms that the manuscript is an honest, accurate, and transparent account of the study being reported; that no important aspects of the study have been omitted; and that any discrepancies from the study as originally planned (and, if relevant, registered) have been explained.

Dissemination to participants and related patient and public communities: The results of this study have been shared with the Medicines and Healthcare products Regulatory Agency (MHRA) which have informed the 2025 Regulating medical devices in the UK regulation. The results have also been shared with the Royal Pharmaceutical Society, General Pharmaceutical Council, Royal College of General Practitioners for dissemination to their members and also have been presented directly to clinicians and researchers at national diagnostics meetings. The work will be disseminated to the general public through a public webinar and through traditional media and social media channels.

Provenance and peer review: Not commissioned; externally peer reviewed.
