# Supplementary material for: Direct-to-consumer self-tests sold in the UK in 2023: cross sectional review of regulation and evidence of performance
Source: BMJ. 2025 Jul 23;390:e085547. doi: 10.1136/bmj-2025-085547 (PMC12284944; doi:10.1136/bmj-2025-085547)
Supplement: Supplementary file 1 — Web appendix: Appendix tables [file hilb085547.ww.pdf]

Direct-to-consumer self-tests sold in the UK in 2023: an investigation into their regulation and the evidence of their performance

Appendix tables and figures

Contents

Table A1. Test characteristics 2

Table A2. Details from clinical study reports and layperson study reports 3

Figure A1. Distributions of characteristics and biomarkers from clinical and layperson study reports 5

Table A3. Assessments of concerns from equipment, sampling, instructions and interpretation 6

Table A4. Details on concerns in test equipment, sampling instructions and documentation 7

Table A5. Readability assessments of IFUs 9

Table A1. Test characteristics

Test ID	Test Product Name	Sample Type	Test type	Biomarker	Manufacturer	UK Distributor	UK stores identified in survey (purchase cost in 2023)	Notified Body
**T1**	Menopause Test	Urine	dipstick	follicle-stimulating hormone	CARE diagnostica Austria	SELFCheck	SuperDrug (£14.49)	0483
**T2**	Flourish Menopause Test Kit	Urine	test strip partially covered in cassette	follicle-stimulating hormone	Veda-lab France	Dendron brands	Tesco (£10.00), Waitrose, Knights Pharmacy, Well Pharmacy	0483
**T3**	Menopause (FSH) Rapid Test	Urine	test strip in cassette	follicle-stimulating hormone	Hangzhou AllTest Biotech China	Suresign	Asda (£4.00)	0123
**T4**	FSH Rapid Menopause Test Midstream	Urine	test strip in cassette	follicle-stimulating hormone	Hangzhou AllTest Biotech China	Newfoundland	Tesco (£10.00)	0123
**T5**	SP-10 Male Fertility Rapid Test	Semen	cassette + cup + syringe + extraction tube with buffer	acrosomal protein SP-10	Hangzhou AllTest Biotech China	Newfoundland	Tesco (£12.50)	0123
**T6**	SpermCheck Fertility	Semen	cassette + cup + bespoke syringe + extraction tube with buffer	acrosomal protein SP-10	PBM Princeton BioMeditech USA	SpermCheck	Boots (£29.99)	1434
**T7**	SwimCount™ Sperm Quality Test	Semen	cup + syringe + bespoke test device*	Progressive Motile Sperm Cells (PMSCs/mL)	MotilityCount ApS Denmark	SwimCount	SuperDrug (£39.99), Well Pharmacy	2797
**T8**	SURE CHECK® HIV Self-Test	Capillary blood	lancet + device + with integrated sample collector and extraction buffer	antibodies to HIV-1 and HIV-2	Chembio Diagnostic System USA	Luas Diagnostics	Boots (£33.95)	0459
**T9**	Female Chlamydia STI Test Kit	Vaginal swab	cassette + sample bottle with extraction buffer	*Not stated*	CARE diagnostica Austria	SELFCheck	SuperDrug (£19.99)	0483
**T10**	Women's Intimate Self-test	Vaginal swab	test device with integrated sample collector	vaginal pH	Biosynex SA France	Boots	Boots (£8.99)	2797
**T11**	Canestest Self-test for Vaginal Infections	Vaginal swab	test device with integrated sample collector	vaginal pH	Peptonic Medical Israel Ltd Israel	Bayer plc	Boots, SuperDrug, ASDA, Morrisons, Sainsbury's, Tesco (£7.50), Knights Pharmacy	0483
**T12**	Urine Infection Test	Urine	dipstick	protein, nitrite and leucocytes	CARE diagnostica Austria	SELFCheck	SuperDrug (£14.49)	0483
**T13**	Bowel Health Test	Faeces	cassette + sample extraction tube and swab with built in buffer	human haemoglobin	CARE diagnostica Austria	SELFCheck	SuperDrug (£14.49)	0483
**T14**	FOB Rapid Test (Faeces)	Faeces	dip stick with sample extraction tube and swab with built in buffer	human haemoglobin	Hangzhou AllTest Biotech China	Newfoundland	Tesco (£10.00)	0123
**T15**	Prostate Health Test	Capillary blood	2 lancets + pipette + cassette + buffer	prostate specific antigen	Veda-lab France	SELFCheck	SuperDrug (£14.99)	0483
**T16**	Stomach Ulcer Test	Capillary blood	2 lancets + pipette + cassette + buffer	helicobacter pylori antibodies	Veda-lab France	SELFCheck	SuperDrug (£14.49))	0483
**T17**	Gluten Sensitivity Test	Capillary blood	2 lancets + pipette + cassette + buffer	anti-tissue transglutaminase IgA antibodies	Veda-lab France	SELFCheck	SuperDrug (£17.49)	0483
**T18**	One step Strep A Swab test	Throat swab (tonsils)	cassette + two buffers + extraction tube	group A streptococcal antigen	Guangzhou Wondfo Biotech China	*Not stated*	Lloyds pharmacy (£14.99)	0123
**T19**	Flowflex™ Influenza A/B Rapid Test (Self-Testing)	Nasal swab	cassette + buffer + extraction tube	Influenza A antigen and Influenza B antigen	ACON Biotech (Hangzhou) China	Newfoundland	Boots, Tesco (£2.50)	0123
**T20**	Flowflex™ SARS-CoV-2 Antigen Rapid Test (Self-Testing)	Nasal swab	cassette + buffer + extraction tube	SARS-CoV-2 nucleocapsid antigen	ACON Biotech (Hangzhou) China	Newfoundland	Boots, Lloyds pharmacy, SuperDrug (3 tests for £5.79), ASDA, Sainsbury's, Tesco, Waitrose, Knights Pharmacy, Well Pharmacy	0123
**T21**	One step test for SARS-CoV-2 Antigen (Colloidal Gold)	Nasal swab	cassette + buffer + extraction tube	SARS-COV-2 antigen	Getein Biotech China	Every Genetic	Sainsbury's, Tesco (£2.00)	1434
**T22**	STEPAHEAD® COVID-19 Antigen Rapid Test Kit (Swab) For Self-Testing	Nasal swab	cassette + buffer + extraction tube	SARS-CoV-2 nucleocapsid protein antigen	Safecare Biotech (Hangzhou) China	SHARE INFO LTD	Lloyds pharmacy (£1.89)	1434
**T23**	Microalbuminuria (MAU) Rapid Test Kit (Colloidal Gold)	Urine	test strip in cassette + colour card	albuminuria	Hangzhou Singclean China	Newfoundland	Tesco (£8.00)	0123
**T24**	TSH Rapid Test Cassette	Capillary blood	lancet + pipette + cassette + buffer	thyroid-stimulating hormone	Hangzhou AllTest Biotech China	Newfoundland	Tesco (£10.00)	0123
**T25**	Ferritin Rapid Test Cassette	Capillary blood	lancet + pipette + cassette + buffer	ferritin	Hangzhou AllTest Biotech China	Newfoundland	Tesco (£8.00)	0123
**T26**	Iron Deficiency	Capillary blood	lancet + pipette + cassette + buffer	ferritin	Hangzhou AllTest Biotech China	Suresign	Asda (£8.00)	0123
**T27**	Vitamin D Rapid Test Cassette	Capillary blood	lancet + pipette + cassette with 2 wells + buffer + colour card	25-hydroxy Vitamin D	Hangzhou AllTest Biotech China	Newfoundland	Tesco (£8.00)	0123
**T28**	Vitamin D Test	Capillary blood	lancet + pipette + cassette with 2 wells + buffer + colour card	25-hydroxy Vitamin D	Hangzhou AllTest Biotech China	Suresign	Asda (£8.00)	0123
**T29**	Cholesterol Level Test	Capillary blood	lancet + blood spot test + colour card	total cholesterol	CARE diagnostica Austria	SELFCheck	SuperDrug (£14.99)	0483
**T30**	Blood Glucose Test	Capillary blood	2 lancets + dipstick + colour card	blood glucose	National Diagnostic Products Australia	SELFCheck	SuperDrug (£12.99)	0123

FOB – faecal occult blood; FSH – follicle-stimulating hormone; HIV – human immunodeficiency virus; MAU – microalbuminuria; STI – sexually transmitted infection; TSH – thyroid-stimulating hormone; USA – United States of America.

Table A2. Details from clinical study reports and layperson study reports

		Clinical Study Report			Layperson Study Report				
**(Test ID) Test Product Name**	**Distributor / Manufacturer**	**Report title<br>Setting, participants and samples**	**Comparator test<br>Blinding**	**Sample size<br>Accuracy metrics**	**Report title<br>Setting, participants and samples**	**Specimen process**	**Comparator test<br>Accuracy assessment**	**Test process**	**Subjective assessments**
**(T4)** FSH Rapid Menopause Test Midstream<br><br>**(T3)** Menopause (FSH) Rapid Test	Newfoundland / Hangzhou AllTest Biotech, China<br><br>Suresign / Hangzhou AllTest Biotech, China	**Clinical Study Report**<br><br>Patients at outpatient services, under emergency treatment or who are hospital inpatients<br><br>No further detail	ABON Biopharm FSH rapid test (mention of ELISA testing being adopted for discrepant results)<br><br>No mention of blinding	N=250<br>85 positive<br>165 negative<br><br>100% sensitivity<br>100% specificity	**Clinical Study Report … By Laypeople**<br><br>N=105<br>Age, education, days since LMP<br>Location of sample: not stated<br>Location of test: not stated	Specimen collection by volunteer.<br><br>Two specimens from each volunteer	ABON FSH rapid test midstream comparator<br>33 positive<br>72 negative<br><br>100% sensitivity<br>100% specificity	1. Volunteer completed 4 tests. Three versions of the index test (midstream, dipstick and cassette) and one of the comparator test performed.<br>2. Judgement of results by volunteer.<br>3. No blinding of assessments.<br>*(Multiple tests performed on multiple sample).*	- General introduction (100%)<br>- Test procedure (100%)<br>- Result interpretation (100%)<br>- Convenience evaluation (99%)
**(T5)** SP-10 Male Fertility Rapid Test	Newfoundland / Hangzhou AllTest Biotech, China	**Clinical Study Report**<br><br>Semen specimens from hospital in Hangzhou, China.<br><br>No further detail	Other sperm SP-10 protein rapid test<br><br>No mention of blinding	N=112<br>60 normal<br>52 abnormal<br><br>98.1% sensitivity<br>98.3% specificity	**Layperson Study Report**<br><br>N=62<br>Volunteers<br>Age, education<br>Location of sample: not stated<br>Location of test: not stated	Specimen collection by volunteer.	Wondfo's SP-10 rapid test<br>4 abnormal<br>58 normal<br><br>100% sensitivity<br>100% specificity	1. Volunteer completed 2 tests. Index test and comparator test.<br>2. Judgement of results by volunteer.<br>3. No blinding of assessments.<br>*(Unclear whether the comparator test is measured on the same sample or a separate sample).*	- General introduction (97%)<br>- Test procedure (100%)<br>- Result interpretation (100%)<br>- Result reading (100%)<br>- Convenience evaluation (97%)
**(T7)** SwimCount™ Sperm Quality Test	SwimCount / MotilityCount ApS, Denmark	**Multi-Center Clinical Study**<br><br>Males attending 3 fertility clinics and donors in 1 sperm bank (Denmark, Spain, Turkey)<br><br>Count per age category provided	Manual sperm count<br><br>No mention of blinding	N=308<br>236 normal<br>72 abnormal<br><br>95.8% sensitivity<br>90.7% specificity	**Evaluation in the hands of lay users**<br><br>N=81<br>Volunteers<br>No descriptions<br>Location of sample: at home<br>Location of test: at home	Specimen collection by volunteer	No comparator<br>66 normal<br>15 abnormal<br><br>No assessment of accuracy	1. Volunteer completed 1 test.<br>2. Judgement of results by volunteer.<br>3. Volunteers uploaded a photo of test which is checked by the laboratory professional.<br>4. No mention of blinding of second assessment.	- Instructions for use (rated 5-4 vs 3-1) (93%)<br>- Applying the semen sample (rated 5-4 vs 3-1) (94%)<br>- Finding the result window (rated 5-4 vs 3-1) (90%)<br>- Interpreting light blue colour (rated 5-4 vs 3-1) (100%)<br>- Interpreting down blue colour (rated 5-4 vs 3-1) (100%)<br>- Determine ease of use (rated 5-4 vs 3-1) (88%)
**(T14)** FOB Rapid Test (Faeces)	Newfoundland / Hangzhou AllTest Biotech, China	**Clinical Study Report**<br><br>No details on participants or samples	ABON FOB rapid test<br><br>No mention of blinding	N=464<br>64 positive<br>400 negative<br><br>98.4% sensitivity<br>99.3% specificity	**Lay User Study Report**<br><br>N=120<br>Volunteers<br>No descriptions<br>Location of sample: not stated<br>Location of tests: AllTest R&D room	Specimen collection by volunteer.<br><br>Two specimens from each volunteer	No comparator<br>4 abnormal<br>58 normal<br><br>100% +ve agreement<br>100% -ve agreement	1. Volunteer completed 2 tests on first sample (dipstick and cassette)<br>2. Two judgements of results by volunteer.<br>3. Professional completed 2 tests on second sample (dipstick and cassette)<br>4. No blinding of assessments.<br>*(Volunteers also tested spiked samples).*	No assessment
**(T23)** Microalbuminuria (MAU) Rapid Test Kit (Colloidal Gold)	Newfoundland / Hangzhou Singclean, China	**Clinical Study Report**<br><br>Samples reclaimed from Nanjing First People's Hospital<br><br>No further detail	Hangzhou Assure Tech microalbuminuria rapid test kit<br><br>No mention of blinding	N=206<br>131 positive<br>75 negative<br><br>98% sensitivity<br>97% specificity	**Laymen study report**<br><br>N=100<br>Volunteer<br>Age, education, gender<br>Location of sample: Not stated<br>Location of tests: Manufacturer R&D laboratory	Specimen collection by 25 volunteers. Quality control spiked specimens tested by 75 volunteers.	No comparator<br>Strip: 22 normal<br>3 abnormal<br>100% +ve agreement<br>100% -ve agreement<br>Cassette: 23 normal<br>2 abnormal<br>100% +ve agreement<br>100% -ve agreement	1. Volunteer completes two tests (strip and cassettes – midstream test not assessed)<br>2. Two judgements of results by volunteer.<br>3. Same sample retested by R&D engineer (strip and cassette)<br>4. No mention of blinding of second assessment.<br>*(75 spiked samples not included: 100% positive agreement and 100% negative agreement).*	- Understood the IFU (100%)<br>- Operated and interpret results (100%)<br><br>*(Laymen who have no reading ability should perform the test after simple explanation by R&D Engineer).*
**(T24)** TSH Rapid Test Cassette	Newfoundland / Hangzhou AllTest Biotech, China	**Clinical Study Report**<br><br>No details on participants or samples	Foresight (ACON) TSH ELISA test kit<br><br>No mention of blinding	N=220<br>54 positive<br>166 negative<br><br>98.1% sensitivity<br>98.2% specificity	**Layperson Study Report**<br><br>N=150<br>Volunteers<br>Age, education<br>Location of sample: not stated<br>Location of test: not stated	Specimen collection by volunteer.<br><br>Unclear how and what samples are taken for the comparator test.	Foresight (ACON) TSH ELISA<br>36 positive<br>112 negative<br><br>100% sensitivity<br>98.2% specificity	1. States both tests performed by volunteer.<br>2. States volunteer completed both tests<br>3. States judgement of results by volunteer.<br>4. No mention of blinding of second assessment.<br>*(However, the ELISA test is a laboratory test and cannot have been run by the volunteer).*	- General introduction (97.3%)<br>- Test procedure (100%)<br>- Result interpretation (100%)<br>- Result reading (98%)<br>- Convenience evaluation assessed (97.3%)
**(T25)** Ferritin Rapid Test Cassette<br><br>**(T26)** Iron Deficiency	Newfoundland / Hangzhou AllTest Biotech, China<br><br>Suresign / Hangzhou AllTest Biotech, China	**Clinical Study Report**<br><br>No details on participants or samples	Cobas E601 CLIA<br><br>No mention of blinding	N=102<br>79 normal<br>23 abnormal<br><br>91.3% sensitivity<br>96.2% specificity	**Clinical Study Report … By Laypeople**<br><br>N=102<br>Volunteers<br>Age, education, gender<br>Location of sample: not stated<br>Location of test: not stated	Specimen collection by volunteer.<br><br>Four specimens from each volunteer	Cobas E601 CLIA<br>79 normal<br>23 abnormal<br><br>91.3% sensitivity<br>96.2% specificity	1. Volunteer complete three tests (3 test lots).<br>2. Three judgements of results by volunteer.<br>3. Does not state who completes the comparator test.<br>4. No mention of blinding of second assessment.	- General introduction (100%)<br>- Test procedure (100%)<br>- Result interpretation assessed (100%)

(Test ID) Test Product Name	Distributor / Manufacturer	Clinical Study Report			Layperson Study Report				
		**Report title**<br>**Setting, participants and samples**	**Comparator test**<br>**Blinding**	**Sample size**<br>**Accuracy metrics**	**Report title**<br>**Setting, participants and samples**	**Specimen process**	**Comparator test**<br>**Accuracy assessment**	**Test process**	**Subjective assessments**
**(T27)** Vitamin D Rapid Test Cassette<br><br>**(T28)** Vitamin D Test	Newfoundland / Hangzhou AllTest Biotech, China<br><br>Suresign / Hangzhou AllTest Biotech, China	**Clinical Study Report**<br><br>No details on participants or samples	Quantitative Vitamin D Test (Rapi-D)<br><br>No mention of blinding	N=90<br>4 deficient<br>56 insufficient<br>30 sufficient<br><br>Deficient >99.9%<br>Insufficient 94.6%<br>Sufficient 93.3%<br>(% agreement in each group)	**Layperson Study Report**<br><br>N=51<br>Volunteers<br>Age, education<br>Location of sample and test in independent laboratory clinical in Italy (to simulate home environment)	First finger-prick specimen collection by volunteer.<br><br>Second venipuncture for comparator test performed by professional.	LC-MS / ECLIA (Roche Cobas e411)<br>2 deficient<br>27 insufficient<br>22 sufficient<br><br>Deficient 100%<br>Insufficient 88.9%<br>Sufficient 100%<br>(% agreement in each group)	1. Volunteer completed tests.<br>2. Judgement of results by volunteer.<br>3. Venipuncture performed by professional.<br>4. Comparator test performed by expert blind to the sampling and self-test results.	- General introduction (86%)<br>- Test procedure (82%)<br>- Result interpretation (100%)<br>- Result reading (94%)<br>- Convenience evaluation assessed (94%)
**(T30)** Blood Glucose Test	SELFCheck / National Diagnostic Products, Australia	**Evaluation Report** *<br><br>Diabetic patients attending outpatient clinic or the Diabetes Centre at City Hospital, Birmingham UK<br><br>No further detail	YSI 2300 analysis<br><br>No mention of blinding<br>*(meter assessed not visual)*	N=120<br>Mean 10.87 mmol/L<br>SD 5.44 mmol/L	**Lay User Evaluation Report**<br><br>N=110<br>Diabetic patients<br>Location of sample and test independent hospital clinic, Australia	First finger-prick specimen collection by volunteer.<br>Second whole blood or plasma sample tested by trained technicians.	YSI 2300 Stat Plus Analyser<br>110 volunteers<br>Correlation 0.99 (p<0.001)	1. Volunteer completed test, comparing test strip colour to a colour card provided.<br>2. Judgement of results by volunteer.<br>3. Comparator test assessed by trained technician.<br>4. No mention of blinding of second assessment.<br>*(Volunteers received training on how to use test strips beforehand).*	No assessment

CLIA – chemiluminescent immunoassays; ELISA – enzyme-linked immunosorbent assay; FOB – faecal occult blood; FSH – follicle-stimulating hormone; IFU – Instructions for Use; LC-MS – liquid chromatography-mass spectrometry; LMP – last menstrual period; MAU – microalbuminuria; R&D – research & development; SD – standard deviation; TSH – thyroid stimulating hormone.

* Note that although this report did not explicitly mention the term 'clinical' in the title, we categorised the evaluation described in this section of the report as a "CSR" based on the information and description provided within the report itself. The comparison between the test strips (in combination with a digital glucose meter) and the YSI 2300 laboratory-based analysis method were described as a "Clinical evaluation" within the report.

Figure A1. Distributions of characteristics and biomarkers from clinical and layperson study reports

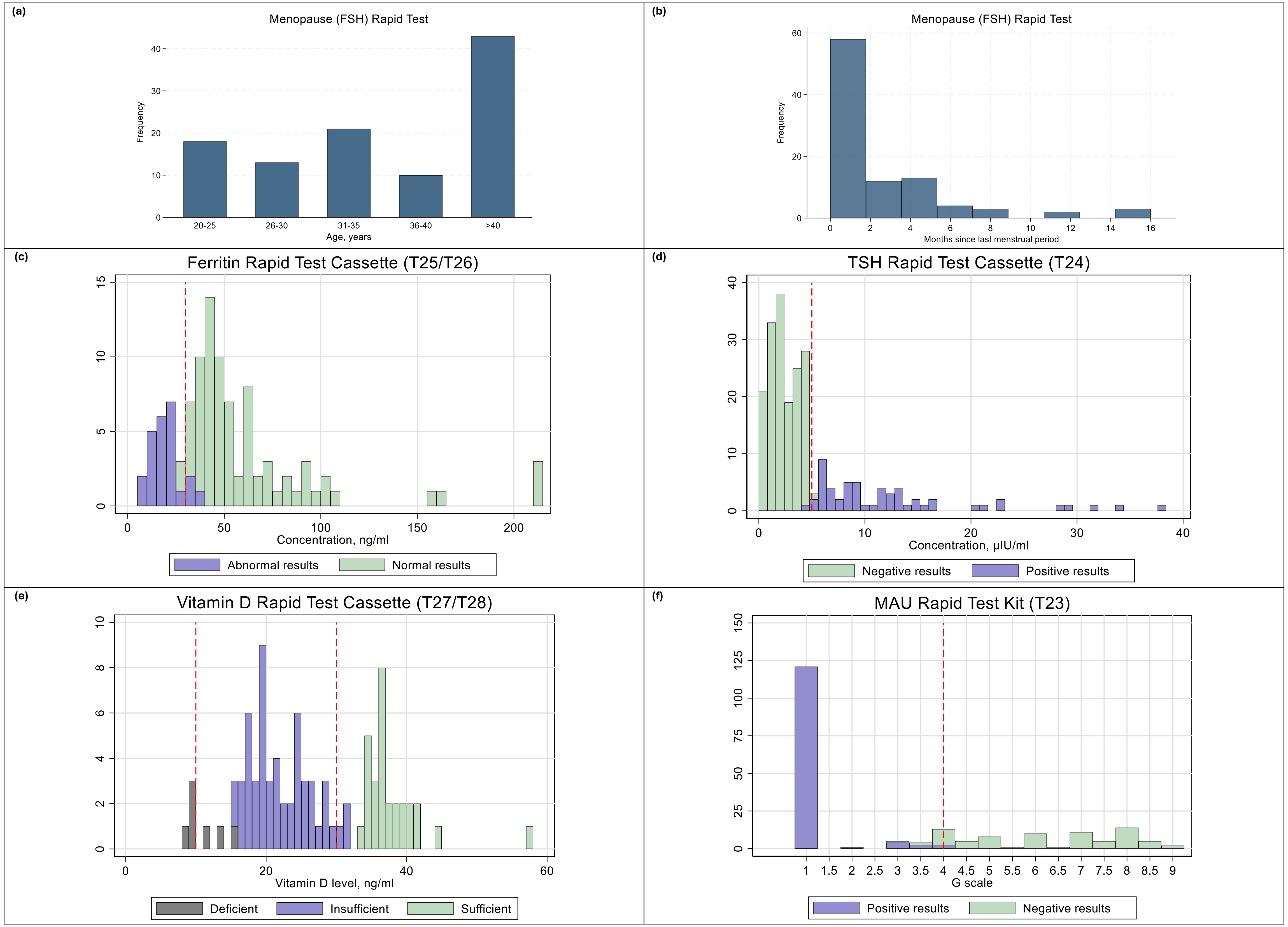

(a) Distribution of age (years) of sample population within LSR evaluating usability of AllTest FSH self-test (T3/T4).

(b) Distribution of months since last menstrual period of sample population within LSR evaluating usability of AllTest FSH self-test (T3/T4).

(c) From FRT self-test (T25/T26) CSR: Stacked histogram showing distribution of CLIA FRT concentration values, with blue representing abnormal AllTest results and green representing normal AllTest results. The dashed line represents the cut-off point of 30ng/ml.

(d) From TSH self-test (T24) CSR: Stacked histogram showing distribution of ELISA TSH concentration values, with blue represented positive AllTest results and green representing negative AllTest results. The dashed line represents the cut-off point of 5µIU/ml.

(e) From Vitamin D self-test (T27/T28) CSR: Stacked histogram showing distribution of vitamin D values measured by the reference standard (Rapi-D quantitative vitamin D test). Grey, blue and green bars represent deficient, insufficient and sufficient levels of vitamin D, respectively, from AllTest results. The dashed lines represent the cut-off points of 10 ng/ml and 30 ng/ml.

(f) From MAU self-test (T23) CSR: Stacked histogram showing distribution of MAU 'G value' results of the reference standard (Assure MAU rapid test). Blue and green bars represent positive and negative results respectively, from the index test (T23). The dashed line represents the cut-off point for a negative result at ≥G4. The G scale (see Fig 1v) appears to represent the strength/boldness of the T line in the test window (higher G scale indicates a bolder line). In this case, both a visible T line and C line indicate a negative test, so a higher G scale indicates a negative test result. Contrary to normal usage (of lateral flow tests) high levels of albuminuria, which is positive, show no T line but only a C line. Lower levels of albuminuria which are negative show a T line and a C line (see Table A4).

MAU – microalbuminuria; CSR – clinical study report; TSH – thyroid stimulating hormone; ELISA - enzyme-linked immunosorbent assay; FRT – ferritin; CLIA - chemiluminescent immunoassay; FSH - follicle-stimulating hormone; LSR – layperson study report.

Table A3. Assessments of concerns from equipment, sampling, instructions and interpretation

Test ID, Test Product Name		Total number of concerns	High-risk concerns			Moderate-risk concerns			Low-risk concerns			Readability	
			Equipment	Sampling	Instructions and interpretation	Equipment	Sampling	Instructions and interpretation	Equipment	Sampling	Instructions and interpretation	Flesch-Kincaid Grade	Font size
T1	Menopause Test	7	2		2	1			1	1		9	9
T2	Flourish Menopause Test Kit	4	1		1				1	1		8	6.5
T3	Menopause (FSH) Rapid Test	8			2	1	1	1	1	1	1	9	6
T4	FSH Rapid Menopause Test Midstream	10			2	1	1	1	1	1	3	9	6
T5	SP-10 Male Fertility Rapid Test	1							1			10	6
T6	SpermCheck Fertility	1									1	8	10
T7	SwimCount™ Sperm Quality Test	3						1	1		1	8	5
T8	SURE CHECK® HIV Self-Test	2	1			1						8	10
T9	Female Chlamydia STI Test Kit	4	1	1		1	1					9	7.5
T10	Women's Intimate Self-test	4						1		3		10	8
T11	Canestest Self-test for Vaginal Infections	3								3		9	6.5
T12	Urine Infection Test	3	1	1	1							10	7.5
T13	Bowel Health Test	4	1		1		2					10	7.5
T14	FOB Rapid Test (Faeces)	7	2		2		2			1		9	6
T15	Prostate Health Test	4	1	1	1				1			8	8
T16	Stomach Ulcer Test	3	1	1					1			8	9
T17	Gluten Sensitivity Test	4	1	1	1				1			8	8
T18	One step Strep A Swab test	4				1	1		2			6	7.5
T19	Flowflex™ Influenza A/B Rapid Test	0										9	7.5
T20	Flowflex™ SARS-CoV-2 Antigen Rapid Test	0										9	7.5
T21	One step test for SARS-CoV-2 Antigen	0										8	6
T22	STEPAHEAD® COVID-19	0										10	5
T23	Microalbuminuria (MAU) Rapid Test Kit	13			5	1	1	2	2		3	10	6.5
T24	TSH Rapid Test Cassette	6	1	1	3	1						9	6
T25	Ferritin Rapid Test Cassette	4		1	2	1						9	6
T26	Iron Deficiency	5		1	3	1						8	6
T27	Vitamin D Rapid Test Cassette	6		1	4	1						9	6
T28	Vitamin D Rapid Test Cassette	6		1	4	1						9	6
T29	Cholesterol Level Test	2				1		1				10	9
T30	Blood Glucose Test	1						1				9	8

FOB – faecal occult blood; FSH – follicle-stimulating hormone; HIV – human immunodeficiency virus; MAU – microalbuminuria; STI – sexually transmitted infection; TSH – thyroid stimulating hormone.

Table A4. Details on concerns in test equipment, sampling instructions and documentation

Test ID	Test product name	Sample type	Equipment	Sampling	Instructions and interpretation
T1	**Menopause Test**	Urine	• No sterile pot provided. (LR)<br>• Required maximum depth of sample not marked on dipstick. (Fig 2i) (MR)<br>• Orientation of dipstick not marked. (Fig 2i) (**HR**)<br>• T and C lines on dipstick not marked. (Fig 2i) (**HR**)	• Unclear whether a midstream urine sample is to be used, and to give appropriate sampling instructions. (LR)	• Results are classified as negative if either when the T line is clear or when the T line shows a line but a different shade of colour from the C line. (**HR**)<br>• Very similar shade of colour for both T and C line. (Fig 2j) (**HR**)
T2	**Flourish Menopause Test Kit**	Urine	• No sterile pot provided. (LR)<br>• T and C result windows are not marked. (Fig 2h) (**HR**)	• Unclear whether a midstream urine sample is to be used, and to give appropriate sampling instructions. (LR)	• Results are classified as negative either when the T line is clear or when the T line shows a line but a different shade of colour from the C line. (**HR**)
T3	**Menopause (FSH) Rapid Test**	Urine	• No sterile pot provided. (LR)<br>• Test cassette was fully encapsulated only leaving 5 slots to touch the urine risking splash onto the reading window. (Fig 2f) (MR)	• Unclear whether a midstream urine sample is to be used, and to give appropriate sampling instructions. (LR)<br>• The figure showing how a urine stream sample should be taken is not anatomically recognisable. (Fig 3a) (MR)	• Instructions state: "Urine specimens exhibiting visible precipitates should be centrifuged, filtered or allowed to settle to obtain a clear specimen for testing". (MR)<br>• Instructions state: "Urine specimens may be stored at 2-8°C (35.6-46.4°F), for up to 48 hours prior to testing". (LR)<br>• The IFU does not give an interpretation of a negative test result in the subgroup who have menopausal symptoms. (**HR**)<br>• Negative results are not clear as they can both be classified as negative if either the T line is absent, or if the T line shows a line but a different shade of colour from the C line. (**HR**)
T4	**FSH Rapid Menopause Test Midstream**	Urine	• No sterile pot provided. (LR)<br>• Test cassette was fully encapsulated only leaving 5 slots to touch the urine risking splash onto the reading window. (Fig 2f) (MR)	• Unclear whether a midstream urine sample is to be used, and to give appropriate sampling instructions. (LR)<br>• The figure showing how a urine stream sample should be taken is not anatomically recognisable. (Fig 3a) (MR)	• Instructions state: "Urine specimens exhibiting visible precipitates should be centrifuged, filtered or allowed to settle to obtain a clear specimen for testing". (MR)<br>• Instructions state: "Urine specimens may be stored at 2-8°C for up to 48 hours prior to testing". (LR)<br>• Instructions state: "For prolonged storage, specimens may be frozen and stored below -20°C". (LR)<br>• Instructions state: "Frozen specimens should be thawed and mixed before testing". (LR)<br>• The IFU does not give an interpretation of a negative test result in the subgroup who have menopausal symptoms. (**HR**)<br>• Negative results are not clear as they can both be classified as negative if either the T line is absent, or if the T line shows a line but a different shade of colour from the C line. (**HR**)
T5	**SP-10 Male Fertility Rapid Test**	Semen	• A 1.0mL volume syringe is provided to collect a 0.1mL sample. (Fig 2n) (LR)		
T6	**SpermCheck Fertility**	Semen			• IFU labels the lateral flow test as positive as normal, whereas the negative as low. (LR)
T7	**SwimCount™ Sperm Quality Test**	Semen	• Result window very small. (LR)		• The shades of blue used to indicate two of the three levels of concentration are similar. (Fig 2t) (MR)<br>• Within the IFU, the definitions of sensitivity and specificity metrics have been interpreted as predictive values. This means that the post-test probability of having a normal sperm count (PPV), given a normal result, is underestimated by 2.6 percentage points (98.6% vs 96% sensitivity), but this also means that the post-test probability of having an abnormal sperm count (NPV), given an abnormal result, is overestimated by 15.2 percentage points (75.8% vs 91% specificity). (LR)
T8	**SURE CHECK® HIV Self-Test**	Capillary	• Dipstick control (C) and test (T) lines are not labelled. (Fig 2s) (**HR**)<br>• No spare lancet. (MR)		
T9	**Female Chlamydia STI Test Kit**	Vaginal swab	• C and T abbreviations not explained in the IFU. (**HR**)<br>• Minimal volume of solution provided. (MR)	• Instructions state to swab through the vagina as far as the neck of the womb, which is challenging, and may affect the reliability of the test. (Fig 3c) (**HR**)<br>• The figure showing where to swab is unclear. (Fig 3c) (MR)	
T10	**Women's Intimate Self-test**	Vaginal swab		• Device to be introduced into the vagina to a fixed depth up to 3cm. (Fig 2r) (LR)<br>• No instructions on moving or rotating. (LR)<br>• Device is inserted for 10 seconds. (LR)	• The differences between pH 4.4 (marked as "normal") and 4.7 to 5 (marked as "higher than normal") are not easy to distinguish. (MR)
T11	**Canestest Self-test for Vaginal Infections**	Vaginal swab		• Device to be introduced into the vagina to a fixed depth up to 5cm. (Fig 2q) (LR)<br>• Instructions to rotate several times. (LR)<br>• No time stated for inserted device. (LR)	
T12	**Urine Infection Test**	Urine	• No sterile pot. (**HR**)	• No indication to use a midstream urine sample to reduce the risk of contamination. (**HR**)	• Very similar shade of colour on dipstick. (Fig 2y) (**HR**)
T13	**Bowel Health Test**	Faeces	• C and T abbreviations not explained in the IFU. (**HR**)	• Three samples are requested which may overfill the device. Only one sample is recommended on the NHS screening committee FIT test. (MR)<br>• The amount of matter that is selected is not clearly described. (MR)	• There is no threshold stated on the test to compare with the NHS screening committee FIT test of 120 µg Hb/g.[1,2] (**HR**)
T14	**FOB Rapid Test (Faeces)**	Faeces	• Dipstick control (C) and test (T) lines are not labelled. (Fig 2k) (**HR**)<br>• The dipstick was marked to a max level in the solution but was not marked as to which end should touch the sample. (Fig 2k) (**HR**)	• Three samples are requested which may overfill the device. Only one sample is recommended on the NHS screening committee FIT test. (MR)<br>• The amount of matter that is selected is not clearly described. (MR)<br>• The sampling illustration is difficult to interpret. (Fig 3e) (LR)	• The claimed threshold of 6 µg Hb/g is lower than the threshold used in the National Screening Committee FIT test of 120 µg Hb/g.[1,2] (**HR**)<br>• The instructions will not be readable during the sample process as they form part of the collection paper which a user tapes onto their toilet seat where they collect the faecal sample. (Fig 3d) (**HR**)
T15	**Prostate Health Test**	Capillary	• It is unclear what the markings "1 2 C" on the results window represent. (**HR**)<br>• Sample well is not marked as 'S' but with an arrow. (LR)	• Pipette does not reliably collect blood which means the sample may be inadequate. (Fig 2a-d) (**HR**)	• No reference ranges provided with which to interpret results. Continuous measurements are needed to interpret this biomarker,[3] which cannot be done using a binary lateral flow test. (**HR**)
T16	**Stomach Ulcer Test**	Capillary	• It is unclear what the markings "1 2 C" on the results window represent. (**HR**)<br>• Sample well is not marked as 'S' but with an arrow. (LR)	• Pipette does not reliably collect blood which means the sample may be inadequate. (Fig 2a-d) (**HR**)	
T17	**Gluten Sensitivity Test**	Capillary	• It is unclear what the markings "1 2 C" on the results window represent. (**HR**)<br>• Sample well is not marked as 'S' but with an arrow. (LR)	• Pipette does not reliably collect blood which means the sample may be inadequate. (Fig 2a-d) (**HR**)	• No reference ranges provided with which to interpret results. Continuous measurements are needed to interpret this biomarker,[4] which cannot be done using a binary lateral flow test. (**HR**)
T18	**One step Strep A Swab test**	Throat swab	• No tongue depressor provided (Fig 3b). (MR)<br>• Sample well not marked as 'S'. (LR)<br>• Requires mixing two buffers with toxic material into an extraction tube. (LR)	• Instructions to swab from the posterior pharynx, tonsils and other inflamed areas whilst not touching the tongue, cheeks and teeth is challenging. (MR)	
T19	**Flowflex™ Influenza A/B**	Nasal swab			

	**Rapid Test (Self-Testing)**				
**T20**	**Flowflex™ SARS-CoV-2 Antigen Rapid Test (Self-Testing)**	Nasal swab			
**T21**	**One step test for SARS-CoV-2 Antigen (Colloidal Gold)**	Nasal swab			
**T22**	**STEPAHEAD® COVID-19 Antigen Rapid Test Kit (Swab) For Self-Testing**	Nasal swab			
**T23**	**Microalbuminuria (MAU) Rapid Test Kit (Colloidal Gold)**	Urine	• No sterile pot. (LR)<br>• Test cassette was fully encapsulated only leaving 3 slots to touch the urine risking splash onto the reading window. (Fig 2g) (MR)<br>• Instructions state using disposable gloves and masks to prevent contact, but no equipment is provided. (LR)	• The figure showing how a urine stream sample should be taken is not anatomically recognisable. (Fig 3a) (MR)	• The threshold concentration is 20 mg/L (usually mg/g). A reference range of 30 mg/g or less is normal; this is likely to over-diagnose. (**HR**)<br>• Instructions state: "If there is sediment at the bottom of the container, please centrifuge or filter". (MR)<br>• Instructions state: "urine specimen can be refrigerated at 2-8 C for 48 hours". (LR)<br>• Instructions state: "For long term storage, specimens should be kept below -20 C". (LR)<br>• Instructions state "Specimens may be infectious or be a potential biological hazard. When collecting another individual urine, wear Disposable gloves and masks to prevent contact". (LR)<br>• A colour card is used to decide whether a value is negative or positive, which has 11 values, with very subtle differences between them. (Fig 2v) (**HR**)<br>• Instructions state: "results are considered positive even if there is a faint line in the T line region, like G3-G4 shown in the colour card included". There are many "faint" lines which makes this assessment ambiguous. (**HR**)<br>• The IFU is confusing regarding whether a positive result is abnormal or normal. Contrary to normal usage (of lateral flow tests), high levels of albuminuria, which is abnormal, show no T line but only a C line. Lower levels of albuminuria, which is normal, show a T line and a C line. (**HR**)<br>• Contrary to normal usage (of lateral flow tests) faint T lines like G3-G4 marked on the colour chart are classified as positive (alongside the test when no T line is seen). (Fig 2v) (**HR**)
**T24**	**TSH Rapid Test Cassette**	Capillary	• The line on the pipette is not marked on the figure (it is marked on all other pipettes used by the same manufacturer, and in the same test sold by a different distributor). (**HR**)<br>• No spare lancet (MR)	• Pipette does not reliably collect blood which means the sample may be inadequate. (Fig 2a-d) (**HR**)	• In the text and figures, steps 6 and 7 are in the opposite order, stating to add buffer first in the figure and to add blood first in the text. These instructions are different in the IFU of the same test marketed by a different distributor. (Fig 3f and 3g) (**HR**)<br>• The text instructions state to use 2 drops of blood, whereas the pipette could hold more blood than this. These instructions are different in the IFU from a different distributor which directs use of the whole pipette full of blood. (Fig 3f and 3g) (**HR**)<br>• No reference ranges provided with which to interpret results. Continuous measurements are needed to interpret this biomarker,[5] which cannot be done using a binary lateral flow test. (**HR**)
**T25**	**Ferritin Rapid Test Cassette**	Capillary	• No spare lancet (MR)	• Pipette does not reliably collect blood which means the sample may be inadequate. (Fig 2a-d) (**HR**)	• No reference ranges provided with which to interpret results. Continuous measurements of are needed to interpret this biomarker,[6 7] which cannot be done in a binary lateral flow. (**HR**)<br>• The IFU states that pregnant women should be tested, but this is likely to lead to overdiagnosis and anxiety as the reference range in pregnant women is very different. (**HR**)
**T26**	**Iron Deficiency**	Capillary	• No spare lancet (MR)	• Pipette does not reliably collect blood which means the sample maybe inadequate. (Fig 2a-d) (**HR**)	• The numbers on the text instructions do not agree with the illustration. (**HR**)<br>• No reference ranges provided with which to interpret results. Continuous measurements are needed to interpret this biomarker,[6 7] which cannot be done using a binary lateral flow test. (**HR**)<br>• The IFU states that pregnant women should be tested, but this is likely to lead to overdiagnosis and anxiety as the reference range in pregnant women is very different. (**HR**)
**T27**	**Vitamin D Rapid Test Cassette**	Capillary	• No spare lancet (MR)	• Pipette does not reliably collect blood which may means the sample is maybe inadequate. (Fig 2a-d) (**HR**)	• No reference ranges provided with which to interpret results. Continuous measurements are needed to interpret this biomarker,[8] which cannot be done in a binary lateral flow. (**HR**)<br>• Interpretation of the abnormal results is confusing as either the presence or absence of the T line could represent an abnormal result. There are three categories which give abnormal results, "deficient", "insufficient" and "excess", with the former two indicated by the presence of a darker T line and the latter indicated by the absence of a T line. (**HR**)<br>• The insufficient section is wrongly marked as white in the colour card. This is different to the colour card from a different distributor of the same test. (Fig 3h and 3i) (**HR**)<br>• The differences in shade of pink between insufficient and sufficient are very subtle. (Fig 2u) (**HR**)
**T28**	**Vitamin D Test**	Capillary	• No spare lancet (MR)	Pipette does not reliably collect blood which means the sample may be inadequate. (Fig 2a-d) (**HR**)	• No reference ranges provided with which to interpret results. Continuous measurements are needed to interpret this biomarker,[8] which cannot be done in a binary lateral flow. (**HR**)<br>• Interpretation of the abnormal results is confusing as either the presence or absence of the T line could represent an abnormal result. There are three categories which give abnormal results, "deficient", "insufficient" and "excess", with the former two indicated by the presence of a darker T line and the latter indicated by the absence of a T line. (**HR**)<br>• The insufficient section is wrongly marked as white in the colour card. This is different to the colour card from a different distributor of the same test. (Fig 3h and 3i) (**HR**)<br>• The differences in shade of pink between insufficient and sufficient are very subtle. (**HR**)
**T29**	**Cholesterol Level Test**	Capillary	• No spare lancet (MR)		• The results can give a mottled pattern without a clear single value. (MR)
**T30**	**Blood Glucose Test**	Capillary			• There are some subtle differences in colour on the colour chart (Fig 2x) (MR)

LR – low risk concern; MR – moderate risk concern; **HR** – high risk concern.
FIT – faecal immunochemical test; FOB – faecal occult blood; FSH – follicle-stimulating hormone; HIV – human immunodeficiency virus; IFU – Instructions for Use; MAU – microalbuminuria; NHS – national health service; NPV – negative predictive value; PPV – positive predictive value; STI – sexually transmitted infection; TSH – thyroid stimulating hormone.
[1] Cancer Research UK. Quality improvements and future optimisation of bowel cancer screening. [updated 07/10/2024. Available from: https://www.cancerresearchuk.org/health-professional/cancer-screening/bowel-cancer-screening/quality-improvements-future-optimisation, accessed Nov 2024].
[2] NICE CKS. Bowel screening: What is the NHS bowel screening programme in the UK? UK: NICE CKS; 2024 [updated Apr 2024. Available from: https://cks.nice.org.uk/topics/bowel-screening/background-information/the-nhs-bowel-screening-programme/, accessed 08/03/2025].
[3] NICE CKS. Prostate cancer: How should I assess a person with suspected prostate cancer? UK: NICE CKS; 2022 [updated Sept 2024. Available from: https://cks.nice.org.uk/topics/prostate-cancer/diagnosis/assessment/, accessed 20/03/2025].
[4] NICE CKS. Coeliac disease: How should I assess a person with suspected coeliac disease? UK: NICE CKS; 2020 [updated May 2020. Available from: https://cks.nice.org.uk/topics/coeliac-disease/diagnosis/assessment/, accessed 11/03/2025].
[5] NICE CKS. Thyroid disease: assessment and management. NICE guideline [NG145]. Recommendations. UK: NICE CKS; 2019 [updated 12 Oct 2023. Available from: https://www.nice.org.uk/guidance/ng145/chapter/recommendations, accessed 11/03/2025].
[6] NICE CKS. Anaemia - iron deficiency: What investigations should I arrange to confirm iron deficiency anaemia? UK: NICE CKS; 2023 [updated Aug 2024. Available from: https://cks.nice.org.uk/topics/anaemia-iron-deficiency/diagnosis/investigations/, accessed 11/03/2025].
[7] The World Health Organization. WHO guideline on use of ferritin concentrations to assess iron status in individuals and populations. Geneva: WHO; 2020 [Available from: https://www.who.int/publications/i/item/9789240000124, accessed 18/02/2025].
[8] NICE CKS. Vitamin D deficiency in adults: When should I suspect or test for vitamin D deficiency? UK: NICE CKS; 2021 [updated Jan 2022. Available from: https://cks.nice.org.uk/topics/vitamin-d-deficiency-in-adults/diagnosis/diagnosis/, accessed 11/03/2025].

Table A5. Readability assessments of IFUs

Test ID	Test name	Number of words	Number of sentences	Number of total syllables	Number of words with 3+ syllables	Percentage % of words with 3+ syllables	Flesch Reading Ease	Flesch-Kincaid Grade	Font size
**T1**	**Menopause Test Kit**	922	55	1420	115	12	61.5	8.8	9
**T2**	**Menopause Test Kit**	1371	115	2190	222	16	61.4	7.7	6.5
**T3**	**Menopause (FSH) Rapid Test**	1734	122	2788	285	16	55.9	9	6
**T4**	**FSH Rapid Menopause Test Midstream**	1582	107	2521	254	16	56.2	9.1	6
**T5**	**SP-10 Male Fertility Rapid Test**	1768	116	2910	316	18	51	9.9	6
**T6**	**Male Fertility Test**	2997	197	4457	320	11	65	8	10
**T7**	**Sperm Quality Test**	1469	89	2123	132	9	69.1	7.7	5
**T8**	**HIV Self Test**	1904	127	2839	236	12	66.1	7.8	10
**T9**	**Female Chlamydia STI Test Kit**	1066	72	1683	162	15	56.3	9.1	7.5
**T10**	**Women's Intimate Self-Test**	1009	66	1619	179	18	52.1	9.8	8
**T11**	**Canestest Self-Test for Vaginal Infections**	1152	95	1899	231	20	52.7	8.9	6.5
**T12**	**Urine Infection Test Kit**	1649	97	2672	245	15	54.8	9.8	7.5
**T13**	**Bowel Health Test Kit**	1345	74	2129	196	15	56.4	9.9	7.5
**T14**	**FOB Rapid Bowel Health Test**	1211	92	2026	206	17	52.5	9.2	6
**T15**	**Prostate Test Kit**	1433	95	2114	162	11	65.3	7.9	8
**T16**	**Stomach Ulcer Test Kit**	1313	108	1961	173	13	65	7.2	9
**T17**	**Gluten Sensitivity Test Kit**	1407	114	2114	194	14	63.3	7.5	8
**T18**	**Strep A rapid self test**	1410	143	2121	198	14	67.3	6.3	7.5
**T19**	**Flowflex Influenza A/B Rapid Test**	1854	151	3100	370	20	50.6	9.3	7.5
**T20**	**Flowflex SARS-CoV-2 Antigen Rapid Test**	1861	162	3080	361	19	51.3	9	7.5
**T21**	**Covid-19 Rapid Self Test Kit**	1457	121	2324	259	18	57.1	8.3	6
**T22**	**COVID-19 lateral flow test**	1881	126	3088	329	17	51	9.9	5
**T23**	**Microalbuminuria Rapid Test Kit**	1124	85	1947	212	19	49.2	9.7	6.5
**T24**	**TSH Thyroid Rapid Test Cassette**	1785	152	2917	305	17	53.5	8.7	6
**T25**	**Ferritin Rapid Test Cassette**	1761	135	2887	313	18	52.4	9.2	6
**T26**	**Iron Deficiency**	1249	110	2016	203	16	57.9	8	6
**T27**	**Vitamin D Rapid Test Cassette**	1825	138	2926	345	19	52.6	9.2	6
**T28**	**Vitamin D Test**	1800	136	2902	328	18	53.9	9	6
**T29**	**Cholesterol Test Kit**	1674	97	2623	223	13	56.7	9.6	9
**T30**	**Blood Glucose Test Kit**	1224	66	1959	119	10	63.5	9	8
**Control documents**									
**Green cross code**		1022	73	1470	85	8	73.8	6.4	
**Harry Potter Book 1**		1337	74	1796	59	4	77.1	7.1	
**Harry Potter Book 7**		1465	94	2155	140	10	71.2	7.2	

FOB – faecal blood occult; FSH – follicle-stimulating hormone; HIV – human immunodeficiency virus; IFU – Instructions for Use; STI – sexually transmitted infection; TSH – thyroid stimulating hormone.
